# Calcineurin inhibition rescues alloantigen-specific central memory T cell subsets that promote chronic GVHD

**DOI:** 10.1172/JCI170125

**Published:** 2024-06-03

**Authors:** Yewei Wang, Md Ashik Ullah, Olivia G. Waltner, Shruti S. Bhise, Kathleen S. Ensbey, Christine R. Schmidt, Samuel R.W. Legg, Tomoko Sekiguchi, Ethan L. Nelson, Rachel D. Kuns, Nicole S. Nemychenkov, Erden Atilla, Albert C. Yeh, Shuichiro Takahashi, Julie R. Boiko, Antiopi Varelias, Bruce R. Blazar, Motoko Koyama, Simone A. Minnie, Andrew D. Clouston, Scott N. Furlan, Ping Zhang, Geoffrey R. Hill

**Affiliations:** 1 Translational Science and Therapeutics Division, Fred Hutchinson Cancer Center, Seattle, Washington, USA.; 2 Department of Hematology, The Second Xiangya Hospital, Central South University, Changsha, China.; 3 QIMR Berghofer Medical Research Institute, Herston, Queensland, Australia.; 4 Faculty of Medicine, The University of Queensland, Brisbane, Queensland, Australia.; 5 Department of Pediatrics, University of Minnesota, Minneapolis, Minnesota, USA.; 6 Envoi Pathology, Brisbane, Queensland, Australia.; 7 Department of Pediatrics and; 8 Division of Medical Oncology, University of Washington, Seattle, Washington, USA.

**Keywords:** Immunology, Transplantation, Bone marrow transplantation, T cells

## Abstract

Calcineurin inhibitors (CNIs) constitute the backbone of modern acute graft-versus-host disease (aGVHD) prophylaxis regimens but have limited efficacy in the prevention and treatment of chronic GVHD (cGVHD). We investigated the effect of CNIs on immune tolerance after stem cell transplantation with discovery-based single-cell gene expression and T cell receptor (TCR) assays of clonal immunity in tandem with traditional protein-based approaches and preclinical modeling. While cyclosporin and tacrolimus suppressed the clonal expansion of CD8^+^ T cells during GVHD, alloreactive CD4^+^ T cell clusters were preferentially expanded. Moreover, CNIs mediated reversible dose-dependent suppression of T cell activation and all stages of donor T cell exhaustion. Critically, CNIs promoted the expansion of both polyclonal and TCR-specific alloreactive central memory CD4^+^ T cells (T_CM_) with high self-renewal capacity that mediated cGVHD following drug withdrawal. In contrast to posttransplant cyclophosphamide (PT-Cy), CSA was ineffective in eliminating IL-17A–secreting alloreactive T cell clones that play an important role in the pathogenesis of cGVHD. Collectively, we have shown that, although CNIs attenuate aGVHD, they paradoxically rescue alloantigen-specific T_CM_, especially within the CD4^+^ compartment in lymphoid and GVHD target tissues, thus predisposing patients to cGVHD. These data provide further evidence to caution against CNI-based immune suppression without concurrent approaches that eliminate alloreactive T cell clones.

## Abstract 2

Calcineurin inhibition mitigates exhaustion and rescues alloreactive central memory T cells in mice following bone marrow transplant.

## Introduction

Allogeneic hematopoietic stem cell or bone marrow transplantation (BMT) is a curative therapy for many hematological diseases and metabolic disorders. However, graft-versus-host disease (GVHD) contributes to considerable morbidity and mortality despite active pharmacological intervention (1, 2). Despite progress in recent decades (2), chronic GVHD (cGVHD) has limited therapeutic options, highlighting the importance of GVHD prevention strategies that invoke immune tolerance. Preclinical murine and human studies have demonstrated a critical role for donor naive T cells (T_N_) in the induction and development of acute and chronic GVHD (3, 4). The ability of T_N_ to induce GVHD is partly due to their high TCR diversity, long-term persistence, and the ability to differentiate into effector and memory T cell populations after BMT (5). T_N_ that escape immune suppression after allogeneic BMT differentiate into stem-like central memory T (T_SCM_) cells, which repopulate the T cell pool and determine the ongoing spectrum of the allogeneic immune response (6, 7). Precursors of exhausted T cells (T_PEX_) are a small subset of memory T cells that retain proliferation potential and are characterized by the expression of *Tcf7*-encoded transcription factor T cell factor 1 (TCF-1), PD-1 and CD62L (8–11). Alloantigen-specific central memory T cells (T_CM_), with or without stem-like properties also contribute to the pathogenesis of GVHD (12–14) due to their capacity for self-renewal and their long-term persistence (15). High expression of CD62L is characteristic of these T cell populations (10) and, thus, a small number of CD62L^+^ T cells have the ability to reconstitute all T cell populations and cause GVHD after adoptive transfer (16). In contrast, alloantigen-primed or alloantigen-specific effector memory T cells (T_EM_) have a limited capacity to expand and mediate GVHD due to cell-intrinsic properties (14, 17). Thus, pharmacological modulation of T cell expansion, differentiation, exhaustion, and persistence may affect the establishment of immune tolerance after BMT.

IL-2 plays a critical role in regulating immune tolerance, effector T cell responses, and the development of immune memory (18). While prolonged exposure to IL-2 signaling promotes terminal differentiation of memory T cells (19), weak IL-2 signaling with enhanced signaling of IL-15 or other common γ-chain cytokines favors development of T_CM_ and T cell longevity (20). Thus, modulation of IL-2 signaling is predicted to have long-term consequences on T cell differentiation and immune tolerance. Activated CD4^+^ T cells are the major producer of IL-2, provide help to CD8^+^ T cells, and are important to the induction of GVHD, particularly when donor and host are MHC-matched (18). Calcineurin inhibitors (CNIs) including Cyclosporine A (CSA) and Tacrolimus (TAC) suppress T cell expansion and function by impairing nuclear factor of activated T cells–mediated (NF-AT–mediated) IL-2 production (21, 22). CNIs are the backbone of standard prophylaxis regimens for acute GVHD (aGVHD) (23). However CSA also impairs the induction of immune tolerance by disrupting central tolerance (24) and homeostasis of regulatory T cells (Tregs) (25). Thus, understanding the effect of CNIs on memory differentiation of alloreactive T cells after BMT and the consequences on long-term immune tolerance represents an important unmet need.

Cyclophosphamide (Cy) is an alkylating agent that acts by forming DNA crosslinks during DNA replication and therefore has a profound effect on dividing cells. T_N_ are thus relatively less sensitive to its actions (6). Cy is generally administered at 3–4 days post-BMT (PT-Cy) and selectively depletes recently stimulated alloreactive effector T cells while sparing regulatory T cell responses (23, 26). PT-Cy has significantly improved the efficacy of GVHD prophylaxis, particularly benefiting haploidentical transplants that has permitted effective BMT across MHC barriers. Hence, we compared the immunomodulatory effect of PT-Cy to CNIs, aiming to provide insight of their respective mechanisms of action.

Here we investigated the mechanisms by which CNIs affect immune tolerance with discovery-based single cell gene expression and T cell receptor (TCR) assays of clonal immunity in tandem with traditional protein-based approaches and preclinical modeling. Collectively, we have shown that although CNIs attenuate aGVHD by preventing the generation and trafficking of effector T cells into target organs, they paradoxically suppress all stages of T cell exhaustion and favor the expansion of alloreactive T_CM_ particularly prominent in the CD4^+^ compartment, preventing the establishment of immune tolerance and promoting cGVHD. These data provide further evidence to caution against CNI-based immune suppression without concurrent approaches that eliminate alloreactive T cell clones.

## Results

### CSA promotes the self-renewal capacity of donor T cells.

**Figure 1:**
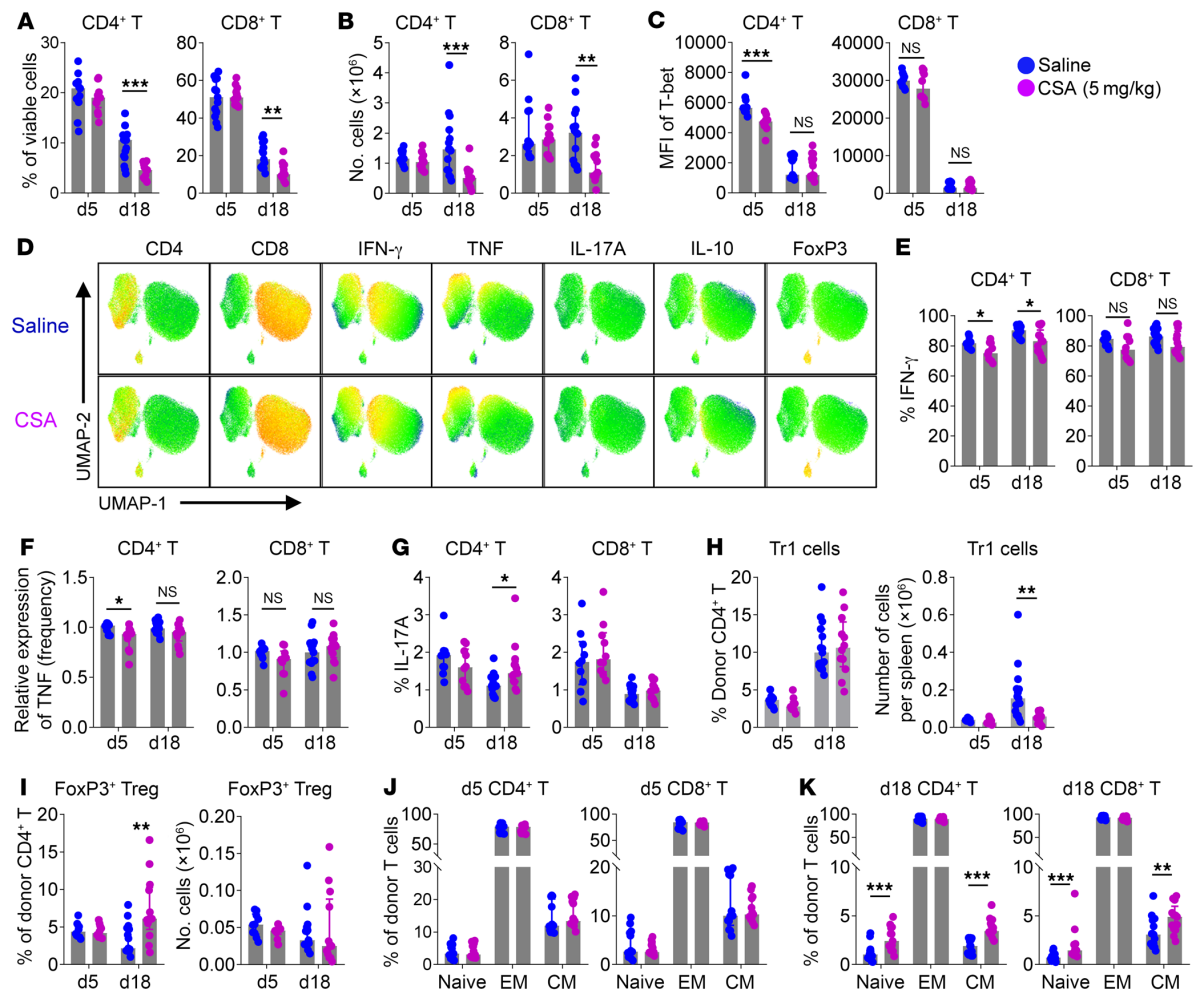
CSA promotes the expansion of central memory donor T cells. B6D2F1 recipients were transplanted with B6 BM (5 × 10^6^) + T cells (2 × 10^6^) and treated with saline or CSA (5 mg/kg/d) up until day 13 after BMT. Spleens were taken 5 or 18 days after BMT and donor CD4^+^ and CD8^+^ T cells were analyzed (*n* = 10–15 per group from 2–3 experiments). (**A**) Frequency of donor T cells in viable cells and (**B**) absolute numbers per spleen. (**C**) Expression of T-bet in donor T cells. (**D**–**H**) Expression of cytokines in donor T cells were analyzed following intracellular cytokine staining: (**D**) UMAP plots of donor T cells on day 5 (concatenated from 5 samples per group), expression of (**E**) IFNγ, (**F**) TNF, (**G**) IL-17A, and (**H**) frequency and numbers of IFNγ^+^IL-10^+^ Tr1 cells. (**I**) Frequency and numbers of CD4^+^ FoxP3^+^ Tregs. (**J**–**K**) Composition of T_N_ (CD44^–^CD62L^+^), T_EM_ (CD44^+^CD62L^–^), and T_CM_ (CD44^+^CD62L^+^) subsets in donor T cells on day 5 (**J**) and day 18 (**K**). Data are presented as median ± interquartile range and analyzed with the Mann-Whitney *U* test. *P < 0.05; **P < 0.01; ***P < 0.001.

CSA is thought to inhibit GVHD by suppressing IL-2 mediated T cell expansion (22, 23). Therefore, we firstly examined the effect of CSA on T cell expansion and function in a preclinical mouse model. We treated mice with CSA at a common clinical weight-based dose (5 mg/kg/d) until day 13 after BMT to mimic the use of CNIs early after clinical BMT, since manifestations of cGVHD can occur by 3 weeks in these murine preclinical systems (27, 28). As expected, CSA inhibited the expansion of donor T cells (Figure 1, A and B) but was associated with only marginal suppression of T-bet and cytokine production that was most consistent in CD4^+^ T cells (Figure 1, C–G). While IFN-γ^+^IL-10^+^ type-1 regulatory (Tr1) T cells (29) were reduced in total number (Figure 1H), FoxP3^+^ Tregs were unaffected (Figure 1I). Given the ability of CSA to modulate IL-2 availability, we next asked if CSA treatment affected the differentiation of memory T cells (19, 20). Indeed, CSA increased the frequency of both CD4^+^ and CD8^+^ T_N_ and T_CM_ subsets beyond 2 weeks after BMT (Figure 1, J and K). Doses of CSA and other therapeutic agents can generally not be interchanged between humans and mice on a per kg basis. Indeed, there have been previous suggestions that body surface area calculations are more appropriate to convert doses across species whereby 5 mg/kg in humans would equate to approximately 60 mg/kg in mice (30), although these assumptions have also been challenged (31). Nevertheless, CSA dosing in mice has generally been between 25 and 100 mg/kg (32–36) and we thus subsequently investigated the effects of higher CSA doses (50 mg/kg/d) to further clarify mechanisms of immune modulation in vivo.

**Figure 2:**
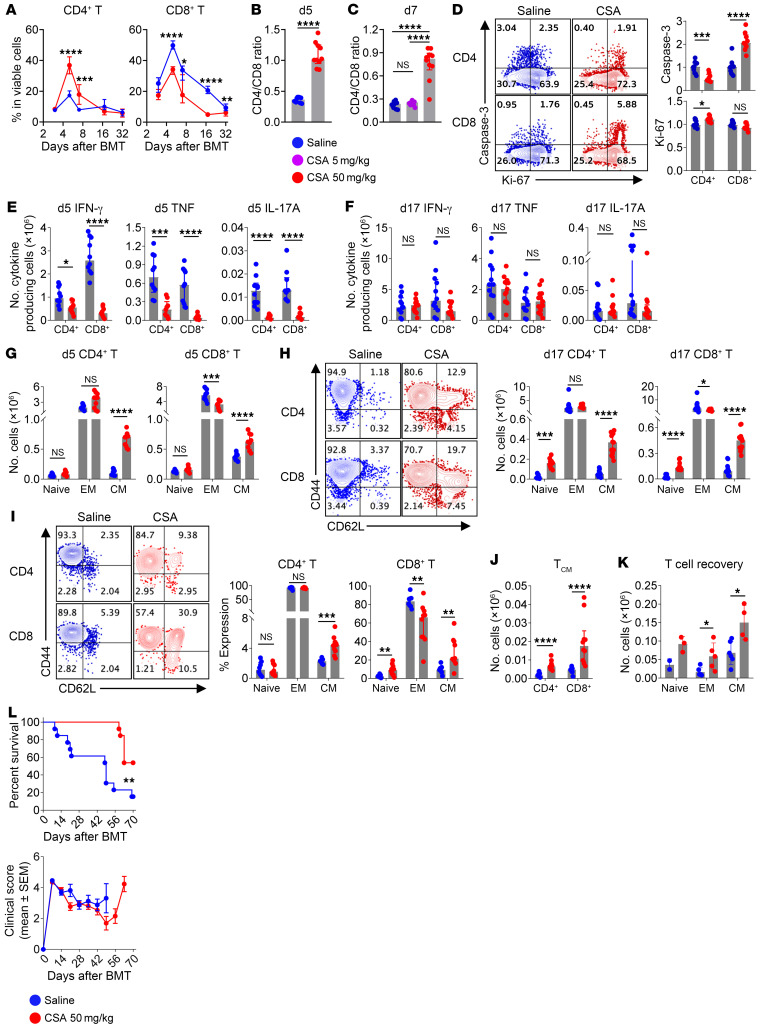
Higher-dose CSA inhibits the acquisition of effector function but promotes survival and central memory differentiation. (**A**–**H**) B6D2F1 recipients were transplanted with B6 BM (5 × 10^6^) + T cells (2 × 10^6^) and treated with saline or CSA (5 or 50 mg/kg/d) until day 13. Spleens were taken for immune phenotyping and donor T cells were gated with congenic markers. (**A**) Frequency of donor T cells in the spleens over time after BMT. (**B** and **C**) Ratio of CD4^+^ to CD8^+^ donor T cells on day 5 (**B**) and day 7 (**C**) (*n* = 10–11 per group from 2 experiments). (**D**) Representative flow cytometric plots and expression of caspase-3 and Ki-67 (relative to the saline group) in donor T cells on day 5 (*n* = 9–10 per group from 2 experiments). (**E** and **F**) Number of cytokine-producing donor T cells on day 5 (*n* = 9–10 per group from 2 experiments) (**E**) and day 17 (**F**) (*n* = 12–13 per group from 3 experiments). (**G** and **H**) Number of donor T_N_ (CD44^–^CD62L^+^), T_EM_ (CD44^+^CD62L^–^), and T_CM_ (CD44^+^CD62L^+^) cells on day 5 (**G**) and day 17 with representative flow cytometric plots (**H**) (*n* = 9–13 per group from 2 experiments). (**I** and **J**) Experiments were set up as described in Supplemental Figure 1I whereby magnetic activated cell sorting–selected (MACS-selected) CD4^+^ and CD8^+^ T cells were combined from saline/CSA-treated mice and transferred to secondary recipients. Memory phenotype (**I**) and numbers of T_CM_ subset (**J**) of the transferred T cells were determined 35 days after transfer (*n* = 8–10 per group from 2 experiments). (**K**) Donor CD4^+^ T cells were isolated from spleens on day 15 after BMT (saline or CSA treated), sort-purified to T_N_, T_EM_ and T_CM_ subsets, and individually transferred to secondary BMT recipients (1 × 10^5^ per recipient) on day 0. Spleens were taken 49 days after adoptive transfer and transferred T cells were determined based on congenic markers (*n* = 2, 3, 6, 5, 6, and 4 per group respectively). (**L**) B6D2F1 recipients were transplanted with B6 BM (5 × 10^6^) + T cells (2 × 10^6^), treated with saline or CSA (50 mg/kg/d) up until day 13 and monitored for survival and clinical scores (*n* = 12–13 per group from 2 experiments). (**A**–**K**) Data are presented as median ± interquartile range and analyzed with the Mann-Whitney U test; (**L**) clinical score data are presented as mean ± SEM and survival analyzed by log rank. *P < 0.05; **P < 0.01; ***P < 0.001; ****P < 0.0001.

Higher doses of CSA significantly suppressed the expansion of CD8^+^ T cells. Conversely, CD4^+^ T cells were increased in frequency and number (Figure 2A and Supplemental Figure 1A; supplemental material available online with this article; https://doi.org/10.1172/JCI170125DS1) resulting in higher CD4^+^-to-CD8^+^ ratios (Figure 2B). These differential effects were only seen at higher CSA doses (Figure 2C and Supplemental Figure 1B). Mechanistically, CD4^+^ T cells had reduced levels of apoptosis after CSA treatment, as determined by caspase-3, with minimal effects on proliferation, as determined by Ki-67. In contrast, higher doses of CSA enhanced apoptosis in CD8^+^ T cells primarily in actively dividing cells (Figure 2D and Supplemental Figure 1C). Cytokine expression was dramatically but reversibly suppressed in both CD4^+^ and CD8^+^ T cells (Figure 2E and Supplemental Figure 1, D and E) by higher dose CSA, which nearly completely recovered following drug withdrawal (Figure 2F and Supplemental Figure 1F). Consistent with our previous findings at lower CSA doses, both CD4^+^ and CD8^+^ T_CM_ were sustained at a higher frequency and numbers in recipients of CSA (Figure 2, G and H, and Supplemental Figure 1, G and H). We next adoptively transferred T cells from BMT recipients receiving CSA treatment into untreated secondary recipients (Supplemental Figure 1I). CSA-treated T cells maintained a higher frequency of T_CM_ 5 weeks after adoptive transfer in both CD4^+^ and CD8^+^ T cell compartments (Figure 2, I and J). Moreover, treatment with CSA enhanced recovery of all memory CD4^+^ subsets (T_CM_ and T_EM_) following adoptive transfer (Figure 2K). Consistently, CSA treatment prevented aGVHD lethality although survivors developed GVHD at later time points beyond 8 weeks (Figure 2L). Therefore, CSA promoted the survival, expansion, and self-renewal capacity of alloreactive T_CM_ but failed to invoke long-term immune tolerance (6, 12).

### CSA differentially inhibits the clonal expansion of CD8^+^ versus CD4^+^ T cells.

**Figure 3:**
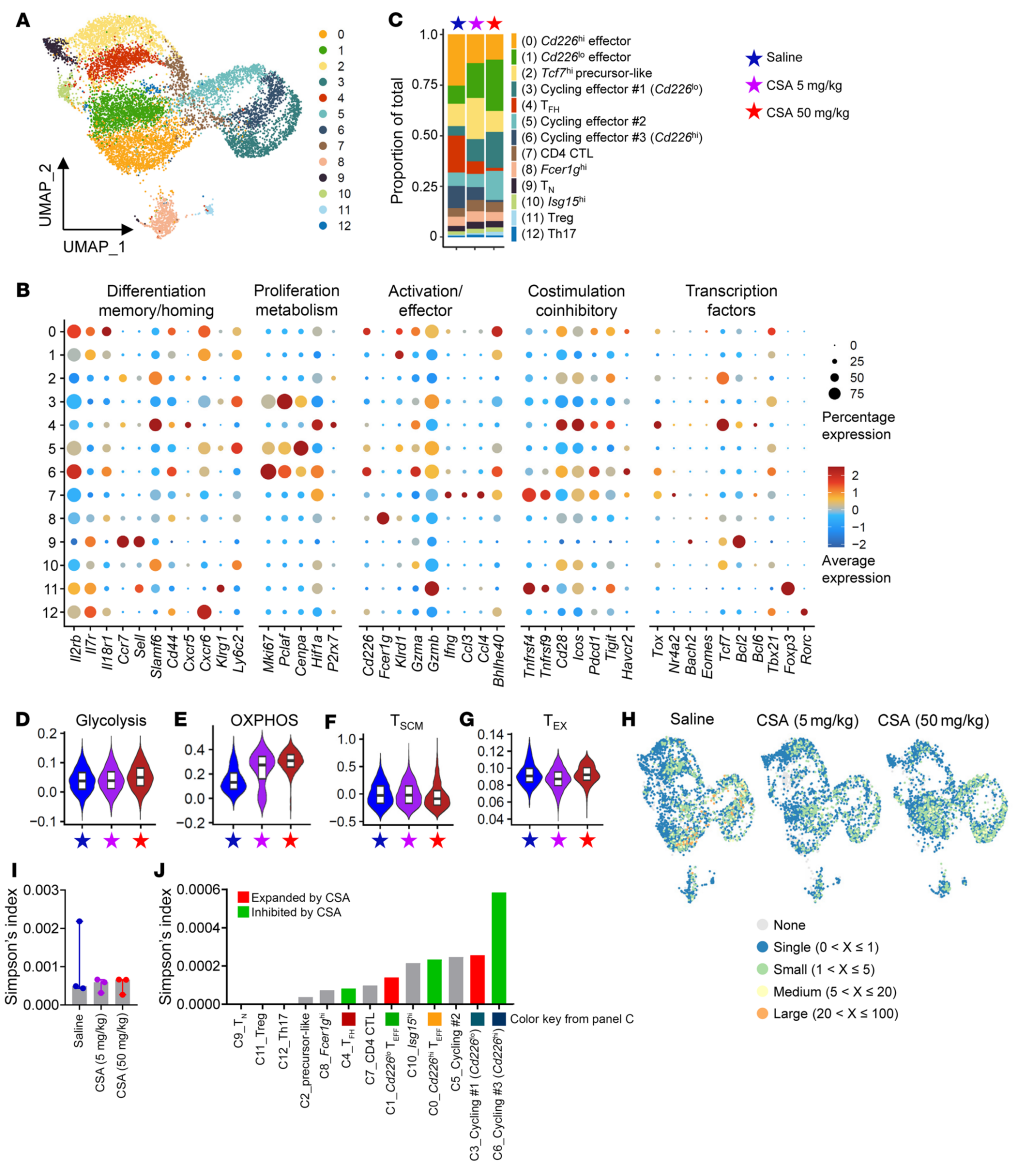
CSA promotes the expansion of quiescent alloreactive CD4^+^ T cells. B6D2F1 recipients were transplanted with B6 BM (5 × 10^6^) + B6 T cells (2 × 10^6^) + TEa TCR transgenic T cells (1 × 10^3^) and treated daily with saline or CSA (5 or 50 mg/kg/kg). Spleens were taken on day 7, pooled from every 2 mice, and donor CD4^+^ T cells were sort purified for single-cell RNA-Seq (*n* = 3 per group). (**A**) UMAP of CD4^+^ T cells colored by clusters. (**B**) Expression of genes across clusters. (**C**) Proportion of each cluster across groups. (**D**–**G**) Gene set enrichment analysis for (**D**) Glycolysis, (**E**) OXPHOS, (**F**) T_SCM_, and (**G**) T_EX_ associated genes across groups. (**H**) UMAP of CD4^+^ T cells colored by clonality scores across groups. (**I**) Simpson’s clonality index of TCR diversity across groups (presented as median ± interquartile range). (**J**) Simpson’s clonality index of TCR diversity across clusters.

We next conducted single cell RNA-Seq on donor T cells 7 days after BMT in which polyclonal WT T cells were transplanted together with alloreactive TEa TCR transgenic T cells (1,000 per recipient). TEa cells (280 cells or 1.3% of total) demonstrated equal distribution across clusters (Supplemental Figure 2A) and therefore were not analyzed separately and were excluded from the analysis of the polyclonal T cell response. We identified 13 clusters in CD4^+^ T cells with clusters 0, 1, 3, 4, and 6 demonstrating differential frequencies across groups (Figure 3, A–C). C0 and C1 were both effector T cells with the latter expressing lower levels of activation and effector RNA (*Cd226*, *Cxcr6*, *Tbx21*, *Gzma*, and *Gzmb*). C3 and C6 were cycling effector T cells (high for *Mki67*, *Pclaf*, *Tbx21*, *Gzma*, and *Gzmb*) with C3 expressing lower activation and effector RNA (*Cd226*, *Cd44*, *Tox*, and *Bhlhe40*) (Figure 3, A–C). C4 expressed follicular helper T cell–associated (T_FH_-associated) RNA (*Bcl6*, *Tcf7*, *Icos*, *Cxcr5*, and *Pdcd1*) (Figure 3, A and B). Of note, CSA quantitatively restrained the activated CD4^+^ T cell clusters (C0, C4, and C6) and expanded the quiescent clusters (C1 and C3) in a dose-dependent manner with upregulation of *Ly6c2* (encoding Ly6C) and *Klrg1* in the latter. The precursor-like cluster (C2) was not expanded by higher dose CSA, however *Ly6c2* and *Sell* were upregulated with concurrent downregulation of activation and effector RNA (*Tigit*, *Pdcd1*, *Tox*, *Gzma*, and *Gzmb*) (Supplemental Figure 2B).

The use of the glycolysis pathway for energy consumption is a hallmark of alloreactive T cells and is critical for the induction of GVHD (37). Consistent with this, the activated clusters (C0, C4, and C6) expressed higher *Hif1a*, a gene highly associated with glycolysis (38). However, the CD4^+^ T cell population as a whole demonstrated limited enrichment for glycolysis-associated genes (Figure 3D). In contrast, oxidative phosphorylation (OXPHOS), an energy generation process mainly used by quiescent T cells (39), was substantially enhanced in CSA-treated groups (Figure 3E), further supporting transcriptomic quiescence. Consistently, the inactive clusters (C1 and C3) demonstrated higher OXPHOS than their nearest activated clusters (C0 and C6) (Supplemental Figure 2, C and D). Interestingly, CD4^+^ T cells demonstrated limited enrichment for T_SCM_ and exhausted T cell–associated (T_EX_-associated) gene sets (Figure 3, F and G), suggesting that CSA differentially modifies T cell function without expanding CD4^+^ T cells with precursor stem cell or exhaustion-associated hallmarks. We next analyzed clonal expansion within CD4^+^ T cell clusters, which revealed low Simpson’s clonality indexes, consistent with high TCR diversity across groups (Figure 3, H and I). The C0, C4, and C6 clusters that were quantitatively inhibited by CSA (Figure 3C) and, in particular, the cycling effectors (C6) had high levels of clonality (Figure 3J). Critically, the quiescent clusters that expanded during CSA treatment (C1 and C3) were also clonal, consistent with the notion that CSA alters the nature of alloreactive CD4^+^ T cell function during GVHD but does not prevent their clonal expansion.

**Figure 4:**
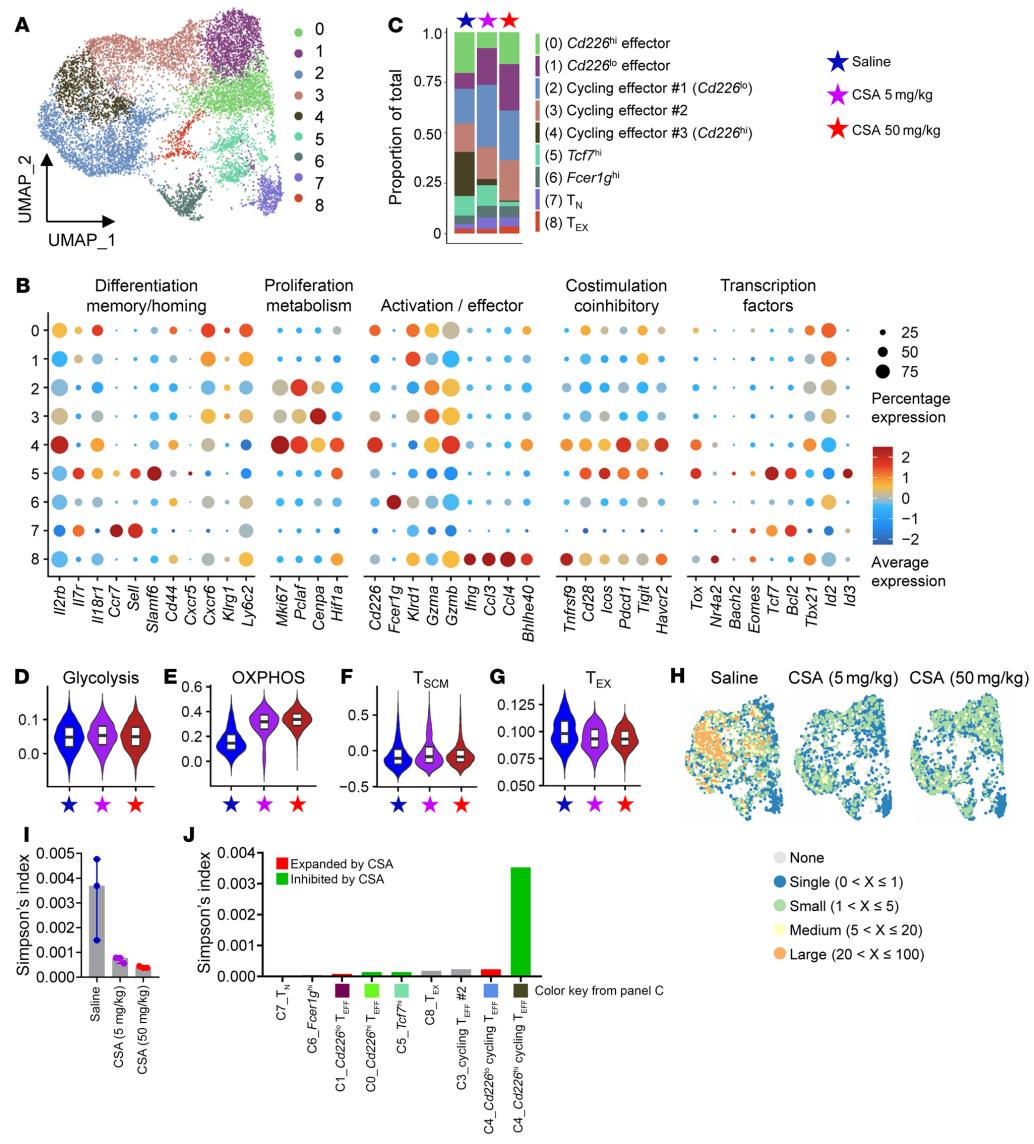
CSA inhibits the clonal expansion in CD8^+^ T cells. Samples were processed as described in Figure 3 and single-cell RNA-Seq was conducted on sort-purified CD8^+^ T cells. (**A**) UMAP of CD8^+^ T cells colored by clusters. (**B**) Expression of genes across clusters. (**C**) Proportion of each cluster across groups. (**D**–**G**) Gene set enrichment analysis for (**D**) Glycolysis, (**E**) OXPHOS, (**F**) T_SCM_, and (**G**) T_EX_ associated genes across groups. (**H**) UMAP of CD8^+^ T cells colored by clonality scores across groups. (**I**) Simpson’s clonality index of TCR diversity across groups (presented as median ± interquartile range). (**J**) Simpson’s clonality index of TCR diversity across clusters.

A similar transcriptomic profile was observed in CD8^+^ T cells in response to CSA. Generally, CSA treatment was associated with a lower number of activated cells (clusters C0 and C4) whereas quiescent cells (clusters C1 and C2) were more numerous (Figure 4, A–C). Similarly, there was limited enrichment for glycolysis-related genes whereas the OXPHOS-related genes were more abundant in mice treated with lower and higher dose CSA (Figure 4, D and E, and Supplemental Figure 3, A and B). CD8^+^ T cells also demonstrated limited expression of T_SCM_ and T_EX_ associated genes, with no expansion seen during CSA treatment (Figure 4, F and G). The C5 cluster was a mixture of T_FH_-like and precursor-like cells (high for *Tcf7*, *Icos*, *Cxcr5*, *Pdcd1*, *Tox*, *Id3*, *Bcl2*, *Sell*, and *Il7r*). The number of cells in this cluster was reduced by higher dose CSA treatment (Figure 4, A–C), but these cells were characterized by increased expression of OXPHOS-related genes (*Atp5e*, *Cox6c*, and *Cox7c*) in addition to *Ly6c2* (Supplemental Figure 3C). Compared with CD4^+^ T cells, a larger proportion of CD8^+^ T cells (C2, C3, and C4) were cycling (Figure 4, A–C) consistent with their more rapid expansion (Supplemental Figure 1B). Unlike CD4^+^ T cells, the degree of CD8^+^ T cell clonality was dramatically lower in CSA treated animals (Figure 4, H and I, and Supplemental Figure 3D), regardless of dose. The cycling effectors (C4) were by far the most clonally expanded CD8 T cell (Figure 4, H and J) and were largely eliminated during higher-dose CSA (Figure 4C). Collectively, CSA treatment downregulated the transcription of activation and effector molecules, inhibited clonal expansion, and upregulated the OXPHOS pathway, consistent with the inhibition of clonal differentiation within CD8^+^ T cells. These generally inhibitory effects of CSA on CD8^+^ T cells are in clear contrast with those seen in CD4^+^ T cells.

### CSA expands CD4^+^ T_CM_ with self-renewal capacity.

**Figure 5:**
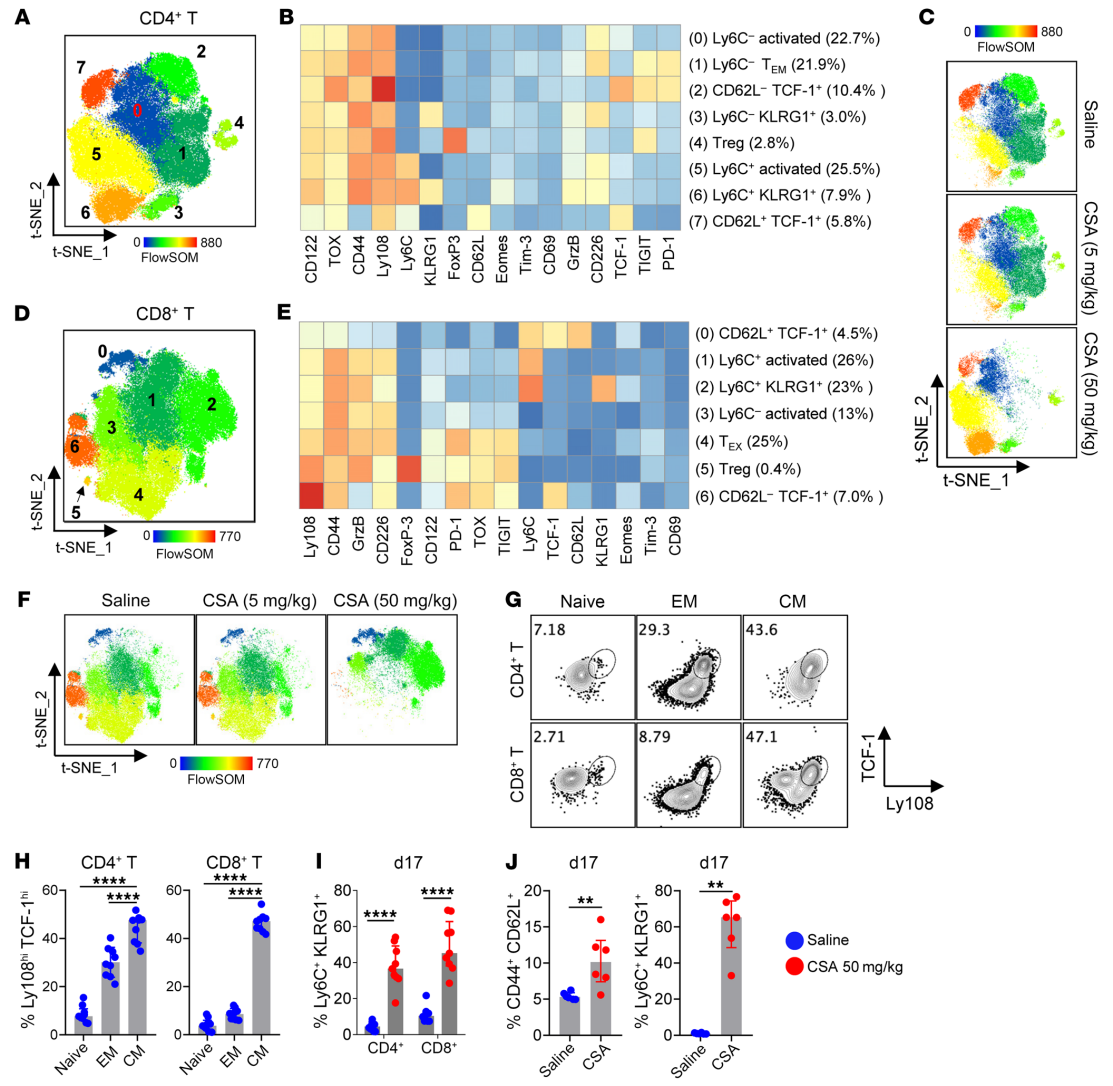
CSA expands alloreactive CD4^+^ T_CM_ with high self-renewal capacity. (**A**–**H**) B6D2F1 recipients were transplanted with B6 BM (5 × 10^6^) + T cells (2 × 10^6^) and treated with saline or CSA (5 or 50 mg/kg/d). Spleens were taken on day 7 and analyzed with high-parameter flow cytometry (concatenated from 4–5 samples per group). (**A**–**C**) Donor CD4^+^ T cells were analyzed for: (**A**) t-SNE plots colored by FlowSOM populations, (**B**) Heatmap of marker expression (MFI) across FlowSOM populations, and (**C**) t-SNE plots (colored by FlowSOM populations) across groups. (**D**–**F**) Donor CD8^+^ T cells were analyzed for: (**D**) t-SNE plots colored by FlowSOM populations, (**E**) Heatmap of marker expression (MFI) across FlowSOM populations, and (**F**) t-SNE plots (colored by FlowSOM populations) across groups. (**G** and **H**) Expression of TCF-7/TCF-1 and Ly108 in T_N_ (CD44^–^CD62L^+^), T_EM_ (CD44^+^CD62L^–^), and T_CM_ (CD44^+^CD62L^+^) subsets of donor T cells in untreated mice (*n* = 9 per group from 2 experiments). (**I**) B6D2F1 recipients were transplanted as above. Spleens were taken on day 17 and analyzed for the memory phenotypes of donor CD4^+^ and CD8^+^ T cells (*n* = 9–10 per group from 2 experiments). (**J**) B6D2F1 recipients were transplanted with B6 TCD BM (5 × 10^6^) + TEa TCR transgenic T cells (5 × 10^3^) and treated with saline or CSA (50 mg/kg/d). Spleens were taken on day 17 and TEa cells were analyzed (*n* = 6 per group from 1 experiment). Data are presented as median ± interquartile range and analyzed with 1-way ANOVA (**H**) or Mann-Whitney U test (**I** and **J**). **P < 0.01; ****P < 0.0001.

We next conducted high-parameter flow cytometry to correlate protein expression with the transcriptomic profiles of donor T cells after BMT. We identified 8 distinct clusters in CD4^+^ T cells (Figure 5, A and B). Clusters 0, 1, 3, 5, and 6 expressed activation markers (CD44, CD226, and TOX) together with various coinhibitory or memory markers (PD-1, TIGIT, Ly6C, and KLRG1), thus representing activated and/or T_EM_. Following higher dose CSA, conventional effector T cell clusters (C1) contracted, whereas alternative effector T cell clusters (C3, C5, and C6), which expressed high levels of Ly6C and KLRG1 and lower levels of TIGIT, were expanded (Figure 5, A–C, and Supplemental Figure 4A). Consistently, CSA induced dose-dependent Ly6C and KLRG1 expression and suppressed CD226, TOX, PD-1, and TIGIT expression (Supplemental Figure 4B). In corroboration with the transcriptomic profiles (Figure 3, A–C), TCF-7/TCF-1^hi^ subsets (C2 and C7) are putatively a mixture of T_FH_ cells and precursor cells, and both clusters are inhibited by higher dose CSA (Figure 5, A–C, and Supplemental Figure 4A). CD8^+^ T cells demonstrated similar phenotypes, such that higher dose CSA significantly decreased the expression of activation and coinhibitory markers (CD226, TOX, PD-1, and TIGIT) with concurrent increase of homing and T cell longevity associated markers (CD62L, Ly6C, and KLRG1) (Figure 5, D–F, and Supplemental Figure 4, B and C). We next examined the T_N_, T_EM_, and T_CM_ subsets for the expression of TCF-7/TCF-1 and Ly108 which are associated with precursor T cells and higher self-renewal ability. Consistent with the enhanced self-renewal ability in T_CM_ (Figure 2, I–K), both CD4^+^ and CD8^+^ T_CM_ demonstrated the highest frequency of Ly108^hi^ TCF-7/TCF-1^hi^ cells (Figure 5, G and H), which have high proliferative potential (10, 11). Furthermore, donor T cells retained high expression of Ly6C and KLRG1 after CSA withdrawal (Figure 5I).

Together these data suggest that CSA may preferentially expand subsets of alloreactive CD4^+^ T cell clones, potentially predisposing recipients to cGVHD following drug withdrawal. To study the effect of CSA on pure alloreactive CD4^+^ T cell clones, we transferred TEa (Ea-specific) TCR transgenic T cells, which respond to recipient alloantigen (Ea peptide from I-E^d^) to undergo clonal expansion (40). Consistent with the data seen in polyclonal T cells, TEa cells demonstrated similar phenotypic changes following higher dose CSA treatment, with enhanced T_CM_ and increased expression of Ly6C and KLRG1(Figure 5J). Collectively, CSA expanded T_CM_ with self-renewal capacity.

### CNIs inhibit all stages of T cell exhaustion and promote the expansion of T_CM_.

**Figure 6:**
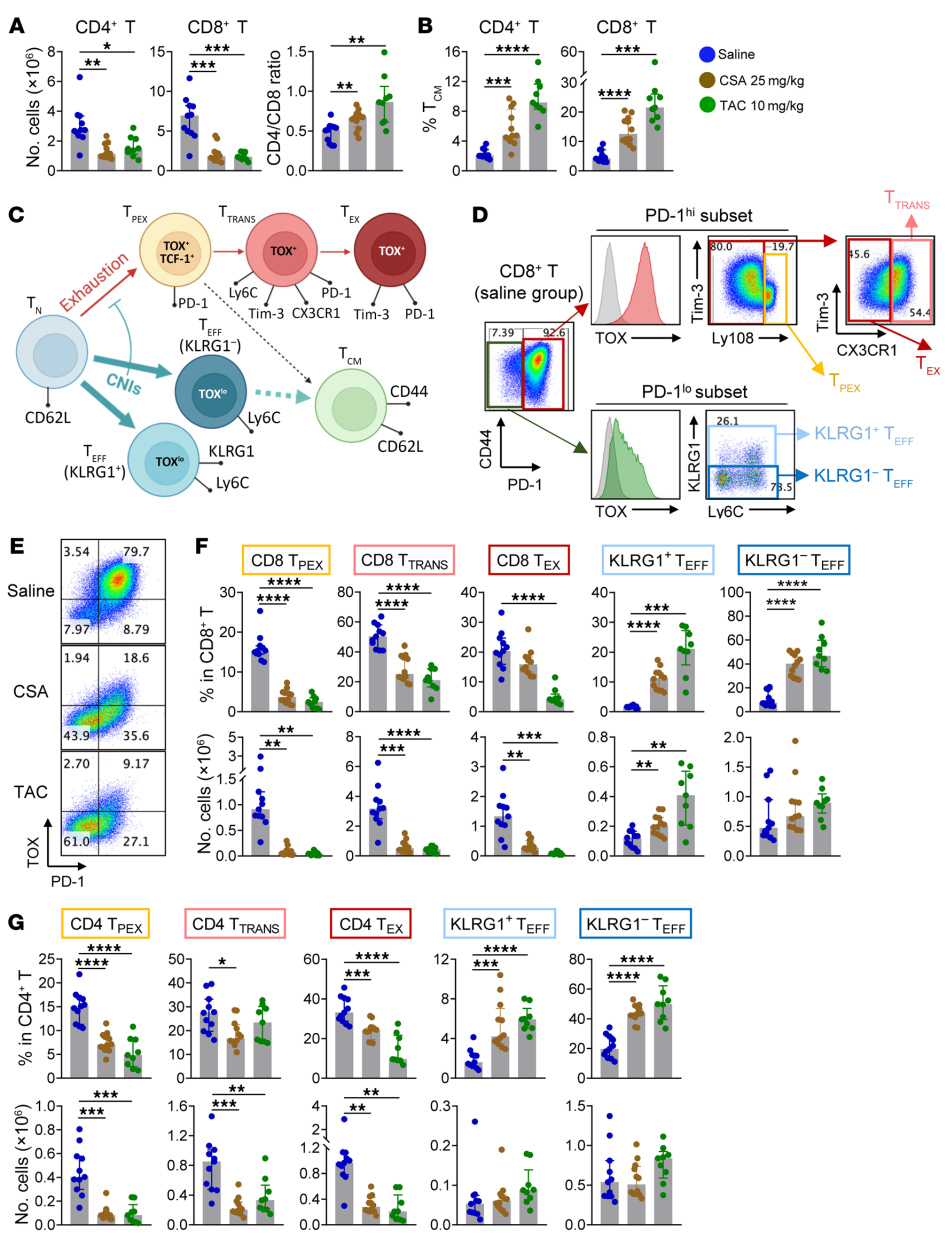
Cyclosporine and Tacrolimus broadly inhibit all stages of T cell exhaustion and promote effector T cell differentiation. Female B6D2F1 recipients were transplanted with B6 BM (5 × 10^6^) + T cells (2 × 10^6^) and treated with saline, CSA (25 mg/kg/d), or TAC (10 mg/kg/d) from day 0 to 13. Spleens were taken on day 14 and analyzed with high-parameter flow cytometry. (**A**) Numbers of donor CD4^+^ and CD8^+^ T cells per spleen with the ratio of CD4^+^ to CD8^+^ donor T cells. (**B**) Frequency of T_CM_ in donor CD4^+^ and CD8^+^ T cells. (**C**) Schema (created with BioRender; biorender.com) of T cell exhaustion and T_EFF_ differentiation pathways whereby CNIs suppress exhaustion (downregulating PD-1 and TOX) and promote T_EFF_ differentiation. (**D**) Gating strategy of the flow cytometric analysis (a representative sample from the saline group) whereby the gray histograms represent fluorescence minus 1 (FMO) control. (**E**) Expression of PD-1 and TOX in CD8^+^ T cells (representative flow cytometric plots). (**F** and **G**) Frequency (upper panels) of the above defined T cell subsets in donor CD8^+^ T cells (**F**) and CD4^+^ T cells (**G**) with corresponding absolute numbers per spleen (lower panels). Data are presented as median ± interquartile range and analyzed with 1-way ANOVA. *P < 0.05; **P < 0.01; ***P < 0.001; ****P < 0.0001.

In a recent study, posttransplant CSA treatment at a dose of 25 mg/kg was said to impair long-term immune tolerance by expanding transitory exhausted donor T cells while suppressing differentiation into the later stages of terminal exhaustion (41). We thus employed high-parameter flow cytometry to further investigate T cell exhaustion and alternative fate differentiation under calcineurin inhibition. We also asked if Tacrolimus (TAC), a widely used CNI in clinical stem cell transplantation, had similar immunomodulatory effects to CSA. We first measured trough levels of CSA and noted that doses of 25 mg/kg resulted in concentrations within or above the standard therapeutic range (Supplemental Figure 5A). In contrast, TAC demonstrated more potent immunomodulatory effects at a dose level of 10 mg/kg (Supplemental Figure 5, B and C) even though the corresponding blood levels were marginally below standard therapeutic ranges. As expected, both agents suppressed T cell expansion, reversed CD4/CD8 ratios and promoted T_CM_ expansion (Figure 6, A and B) consistent with the phenotypes induced by CSA at 50 mg/kg.

We next examined described states of exhaustion within CD8^+^ T cells whereby the expression of the transcription factor TOX is the hallmark of all T cells within the exhaustion pathway (42–46). The exhausted population (PD-1^hi^ TOX^hi^) was further divided into 3 well-defined subsets including precursors of exhausted T cells (T_PEX_, Ly108^hi^), transitory exhausted T cells (T_TRANS_-like, Ly108^lo^ CX3CR1^hi^), and T_EX_ (Ly108^lo^ CX3CR1^lo^; a combination of intermediately and terminally exhausted subsets) as depicted in Figure 6, C and D (47–49). Effector T cells (T_EFF_, PD-1^lo^ TOX^lo^ CD44^hi^) were further divided into KLRG1^–^ T_EFF_ and KLRG1^+^ short-lived T_EFF_. Both CSA and TAC substantially downregulated PD-1 and TOX (Figure 6E), whereas CD44 expression was not affected, consistent with the broad inhibition of all exhausted T cell subsets and concurrent expansion of T_EFF_ (Figure 6F). Of note, transitory T_EX_ were not selectively expanded by CNI administration. We next applied the same gating strategy to CD4^+^ T cells and noted similar results (Figure 6G). Interestingly, while TCF-1/TCF-7 expression was inhibited in T_EM_, T_CM_ retained expression despite downregulation of PD-1 (Supplemental Figure 5D); thus, CD62L^+^ central memory T cells represent the major population of TCF-1/TCF-7^+^ T cells (10, 11). We also noted that the PD-1^lo^ T_EFF_ subsets and not the T_TRANS_-like subset demonstrated the highest expression of Ly6C. Further, CNIs broadly upregulated Ly6C expression such that Ly6C did not discriminate any specific T cell subset (Supplemental Figure 5, E and F) and is therefore not a reliable marker for transitory T_EX_ cells, as has been previously suggested (41). Thus, CNIs suppress all stages of the alloantigen-driven exhaustion pathway, do not expand transitory exhausted T cell subsets, and, instead, promote memory donor T cell expansion, correlating with upregulation of Ly6C, KLRG1, and expansion of alloreactive T_CM_ (summarized in Figure 6C). This process profoundly impairs the induction of immune tolerance. Further, CNI treatment inhibited CD4^+^ FoxP3^+^ Tregs (Supplemental Figure 5G), representing additional contributors to the inhibition of tolerance.

We also examined the contribution of IL-15 signaling to the CNI-expanded T_CM_ differentiation since CNI-mediated IL-2 deprivation promoted the expression of the IL-2Rβ (Supplemental Figure 6A) that is a shared component of IL-2 and IL-15 receptors (IL-2R and IL-15R). However, the transplantation of *Il15ra^–/–^* donor T cells did not disrupt CNI-induced T_CM_ expansion (Supplemental Figure 6B). Thus other cytokines such as IL-7 are likely to be responsible for this effect and this requires further investigation.

### CSA promotes the survival and expansion of alloantigen-specific CD4^+^ T cells.

**Figure 7:**
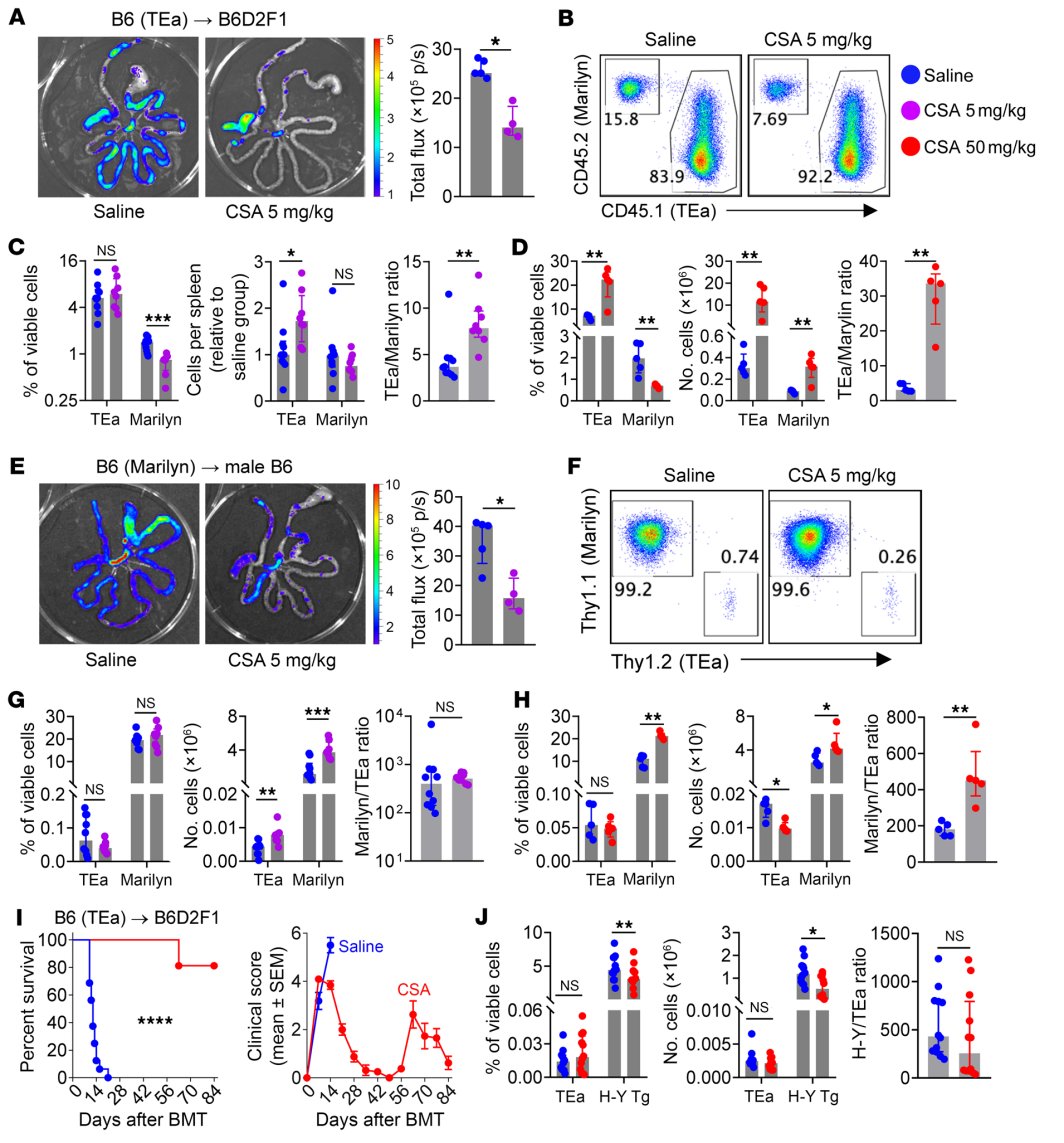
CSA attenuates acute GVHD but promotes alloantigen-specific CD4^+^ T cell survival. (**A**–**D**) Female B6D2F1 (I-E^d^) recipients were transplanted with 5 × 10^6^ PTPrca TCD BM, 5 × 10^4^ luciferase-expressing TEa^luc+^ T cells and 6 × 10^5^ Marilyn T cells, treated with saline or CSA (5 or 50 mg/kg/d) and taken for analysis on day 7. (**A**) Representative bioluminescence images and total flux of gut infiltrating TEa^luc+^ cells (*n* = 4–5 per group). (**B**) Representative flow cytometric plots and (**C** and **D**) frequencies, numbers and ratio of transferred T cells in spleens treated with lower dose CSA (5 mg/kg/d) (**C**: *n* = 8–10 per group from 2 experiments) or with higher dose CSA (50 mg/kg/d) (**D**: *n* = 5 per group). (**E**–**H**) Male B6 recipients were transplanted with 5 × 10^6^ C57xPTP TCD BM, 5 × 10^4^ TEa T cells and 6 × 10^5^ luciferase-expressing Marilyn^luc+^ T cells, treated with saline or CSA (5 or 50 mg/kg) and taken for analysis on day 7. (**E**) Representative bioluminescence images and total flux of gut infiltrating Marilyn^luc+^ T cells (*n* = 4–5 per group). (**F**) Representative flow cytometric plots and (**G** and **H**) frequencies, numbers, and ratio of transferred T cells in spleens treated with lower dose CSA (5 mg/kg/d) (**G**: *n* = 8–10 per group from 2 experiments) or with higher dose CSA (50 mg/kg/d) (**H**: *n* = 5 per group). (**I**) B6D2F1 recipients were transplanted with B6 TCD BM (5 × 10^6^) and 1 × 10^4^ TEa T cells, treated with saline or higher dose CSA (50 mg/kg/d) and monitored for survival and clinical scores (*n* = 16 per group from 2 experiments). (**J**) Male B6 recipients were transplanted with 5 × 10^6^ B6 TCD BM, 5 × 10^4^ TEa T cells and 1 × 10^6^ H-Y specific CD8^+^ T cells and treated with saline or CSA (50 mg/kg/d). Spleens were taken on day 7 (*n* = 12 per group from 2 experiments) and analyzed for the frequency, numbers, and ratio of transferred T cells. (**A**–**H**, and **J**) Data are presented as median ± interquartile range and analyzed with the Mann-Whitney U test; (**I**) Survival data are analyzed with log-rank test, and clinical scores are presented as mean ± SEM. *P < 0.05; **P < 0.01; ***P < 0.001; ****P < 0.0001.

Having established the effect of CNIs in inducing functional and transcriptomic quiescence and suppressing exhaustion while promoting donor T_CM_ expansion, we next sought to delineate the effect of CSA on alloantigen-specific T cells and GVHD with a focus on the CD4^+^ compartment. TEa and Marilyn (H-Y–specific) TCR transgenic T cells were cotransferred into female B6D2F1 recipients. In this system, TEa^luc+^ cells (expressing luciferase) are activated by recipient-derived alloantigen, while Marilyn cells are bystander nonalloreactive CD4^+^ T cells that control for homeostatic proliferation within lymphodepleted recipients. Lower dose CSA (5 mg/kg/d) effectively suppressed the expansion of TEa^luc+^ cells in the intestine, as determined by bioluminescence imaging (Figure 7A). However, TEa cells were more resistant to CSA inhibition in lymphoid organs, resulting in a higher TEa-to-Marilyn ratio (Figure 7, B and C), which was further amplified by higher dose CSA (Figure 7D). We confirmed this effect in a second system whereby Marilyn^luc+^ cells (expressing luciferase) were activated by recipient H-Y male antigen, whereas TEa cells underwent homeostatic expansion in syngeneic recipients (50). CSA suppressed the expansion of Marilyn^luc+^ cells in the intestine (Figure 7E) and again increased the ratio of antigen specific T cells (Marilyn) to control T cells (TEa) in secondary lymphoid organs (Figure 7, F–H). Hence, CSA prevented alloantigen specific CD4^+^ T cell infiltration into GVHD target tissues but was permissive of their accumulation within lymphoid organs. Consequently, higher dose CSA prevented the aGVHD induced by TEa transgenic CD4^+^ T cells, but survivors developed cGVHD following withdrawal of CSA at day 13 (Figure 7I). In contrast to CD4^+^ T cells and consistent with our data of polyclonal CD8^+^ T cells, expansion of H-Y antigen–specific TCR transgenic CD8^+^ T cells were inhibited by CSA (Figure 7J). Collectively, CSA attenuated aGVHD during treatment, but paradoxically rescued alloantigen specific CD4^+^ T cells in the peripheral lymphoid organs, thus actively preventing the establishment of long-term immune tolerance.

### CSA and posttransplant cyclophosphamide mediate differential effects on alloreactive T cells.

**Figure 8:**
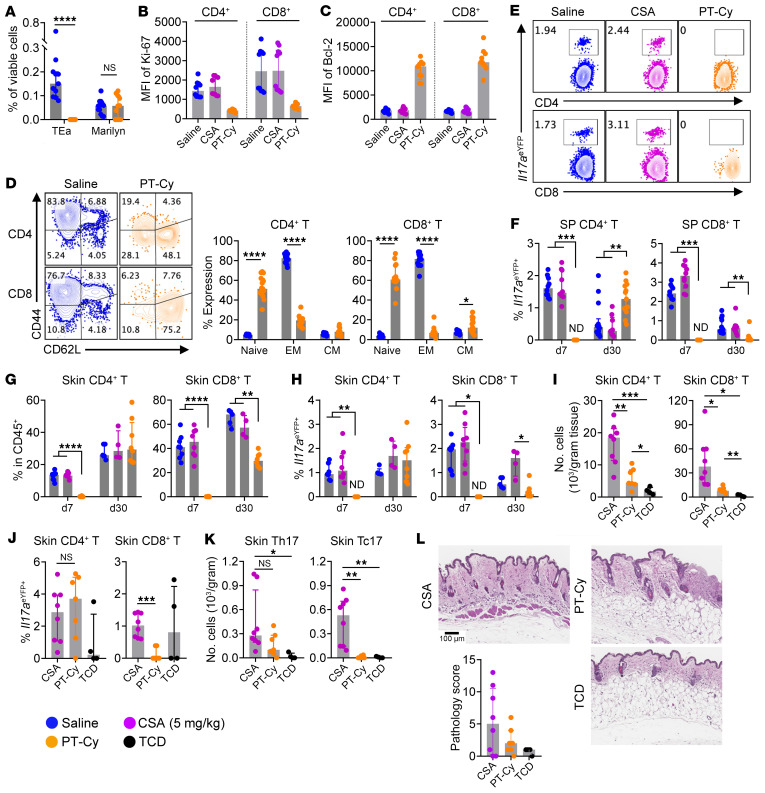
CSA and PT-Cy have differential effects on cGVHD effector pathways. (**A**) Female B6D2F1 recipients were transplanted with 5 × 10^6^ C57xPTP TCD BM, 2 × 10^3^ TEa T cells, and 4 × 10^5^ Marilyn T cells and treated with saline or PT-Cy. Spleens were taken on day 30 (*n* = 11 per group from 2 experiments) and analyzed for the expansion of transferred T cells. (**B**–**H**) B6D2F1 recipients were transplanted with BM (5 × 10^6^) and T cells (2 × 10^6^) from B6.*Il17a*^eYFP^ donors, and treated with saline, CSA (5 mg/kg), or PT-Cy (100 mg/kg). (**B**–**D**) Spleens were taken on day 7 and donor T cells were analyzed for: (**B**) Expression of Ki-67, (**C**) Expression of Bcl-2, and (**D**) composition of T_N_ (CD44^–^CD62L^+^), T_EM_ (CD44^+^CD62L^–^), and T_CM_ (CD44^+^CD62L^+^) subsets. (**E** and **F**) Spleens were analyzed for: (**E**) representative flow cytometric plots of *Il17a*^eYFP^ in donor T cells on day 7 with (**F**) frequencies on day 7 and 30 (*n* = 10–14 per group from 2–3 experiments). (**G** and **H**) Mononuclear cells were isolated from skin and analyzed for: (**G**) frequency of donor T cells with (**H**) *Il17a*^eYFP^ expression on day 7 (*n* = 8 per group from 2 experiments) and day 30 (*n* = 4–8 per group from 1 experiment). (**I**–**L**) B6D2F1 recipients were transplanted with BM (5 × 10^6^) + T cells (2 × 10^6^) or TCD BM (5 × 10^6^) from B6.*Il17a*^eYFP^ donors. BMT recipients were treated with CSA (5 mg/kg/d for 14 days) or PT-Cy. Skin was taken on day 56 for analysis (*n* = 8 for CSA, 7 for PT-Cy, and 4 for TCD groups respectively): (**I**) numbers of donor T cells per gram skin tissue, (**J**) expression of *Il17a*^eYFP^ in donor T cells, (**K**) numbers of Th17 and Tc17 cells per gram skin tissue, and (**L**) representative images of H&E staining with pathology scores. Data are presented as median ± interquartile range and analyzed with the Mann-Whitney *U* test (**A**), 2-way ANOVA (**D**), or 1-way ANOVA (**F**–**K**). ND, not detectable; *P < 0.05; **P < 0.01; ***P < 0.001; ****P < 0.0001.

We further investigated the effect of CSA on alloreactive T cells and its consequences on long-term immune tolerance by comparing CSA with posttransplant cyclophosphamide (PT-Cy). We first tested the effect of PT-Cy on alloantigen specific T cells by transplanting female B6D2F1 recipients with donor CD4^+^ TEa (alloantigen-specific) and Marilyn (bystander) T cells. While a small dose of CD4^+^ TEa cells (2,000 per recipient) were completely deleted by PT-Cy, Marilyn T cells persisted (Figure 8A). The selective deletion of alloreactive T cells by PT-Cy was further investigated using polyclonal donor T cells. As expected, PT-Cy effectively deleted donor T cells early after BMT (Supplemental Figure 7, A and B) along with suppression of cytokine production (Supplemental Figure 7, C–F). We observed lower Ki-67 (Figure 8B) and higher Bcl-2 (Figure 8C) in T cells that survived PT-Cy. This was consistent with the fact that dividing cells were effectively targeted by PT-Cy, whereas T_N_ survived (Figure 8D) to reconstitute the donor T cell pool (6, 51). PT-Cy thus invoked sustained suppression of polyclonal T cells (Supplemental Figure 7G) relative to CSA. Consistent with previous reports (52), PT-Cy was associated with increases in CD4^+^FoxP3^+^ Tregs (29) but decreases in FoxP3^–^ IL-10^+^ Tr1 cells (Supplemental Figure 7, H–J).

We and others have demonstrated the critical role of IL-17A–producing cells in the development of cGVHD (27, 53, 54). We therefore examined the differentiation of IL-17A-producing CD4^+^ (Th17) and CD8^+^ (Tc17) T cells, which contribute to the development of fibrotic acute and chronic GVHD, especially in the skin (27, 55). PT-Cy demonstrated sustained suppression on Tc17 both in the spleen and skin (Figure 8, E–H). In contrast, CSA did not have significant effects on either Th17 or Tc17 populations. We further investigated if this differential effect on Tc17 cells was associated with protection from the development of skin GVHD late (at 8 weeks) after BMT. Indeed, PT-Cy resulted in significantly lower infiltration of Tc17 cells into the skin (Figure 8, I–K) and prevented severe skin cGVHD, as measured by pathology scores over 5 (Figure 8L). Collectively, CSA and PT-Cy thus mediate differential effects on alloreactive T cells after allogeneic BMT resulting in the prevention versus induction of long-term immune tolerance, respectively.

### CSA-expanded CD4^+^ T_CM_ mediate late-onset GVHD.

**Figure 9:**
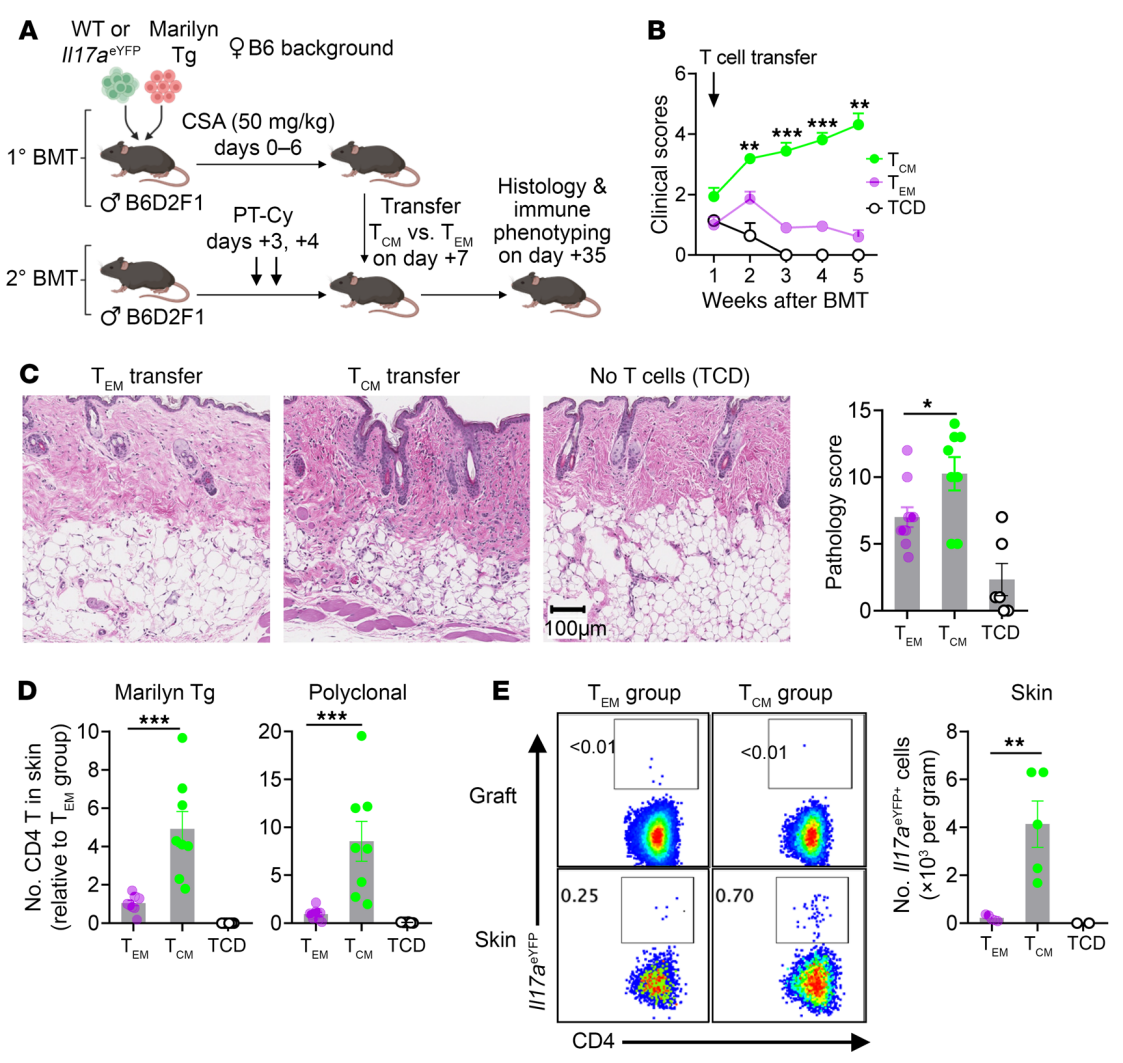
CSA-expanded T_CM_ preferentially mediate late GVHD. Male B6D2F1 recipients were transplanted with 5 × 10^6^ B6 TCD BM (CD45.2 or CD45.1), 1 × 10^6^ B6 CD4^+^ T cells (WT or *Il17a*^eYFP^, CD45.2, CD90.2), and 0.15 × 10^6^ Marilyn CD4^+^ TCR Tg T cells (CD45.2, CD90.1) followed by CSA (50 mg/kg) from day 0. Spleen and mesenteric lymph nodes were isolated on day +7 and sorted as CD4^+^ T_EM_ and T_CM_. Equal numbers of sort-purified CD4^+^ T_EM_ and T_CM_ (5 × 10^5^ per mouse) were adoptively transferred into secondary BMT recipients (male B6D2F1) of a parallel experiment which had been transplanted with TCD BM. Clinical scores were undertaken weekly and analysis was conducted at 28 days after T cell transfer. (**A**) Experimental schema (created with BioRender; biorender.com). (**B**) Clinical scores. (**C**) Pathology scores in the skin and representative images. (**D** and **E**) Adoptively transferred T cells were identified by congenic markers following enzymatic digestion of the skin tissue. (**D**) Numbers of transferred Marilyn Tg and polyclonal CD4^+^ T cells (relative to the T_EM_ group). (**E**) Numbers of *Il17a*^eYFP+^ polyclonal CD4^+^ T cells in the skin with concatenated flow cytometric plots in the skin and the graft. (**B**–**D**) *n* = 10, 8, and 6 per group from 2 experiments; (**E**) *n* = 5, 5, and 2 per group from 1 experiment. Data are presented as mean ± SEM and analyzed with 2-way ANOVA (**B**) or 2-tailed *t* test (**C**–**E**). *P < 0.05; **P < 0.01; ***P < 0.001.

To further investigate the effect of T_CM_ on the pathogenesis of late-onset GVHD, we adoptively transferred CSA-expanded CD4^+^ T_EM_ and T_CM_ to secondary BMT recipients. In this system, T_EM_ and T_CM_ were generated from polyclonal and Marilyn Tg CD4^+^ T cells after primary allogeneic BMT in the presence of CSA, and then were transferred to secondary BMT recipients (Figure 9A). Compared with CD4^+^ T_EM_, the transfer of CD4^+^ T_CM_ resulted in higher clinical scores (Figure 9B) and more severe cutaneous GVHD pathology (Figure 9C). The transfer of CD4^+^ T_CM_ was associated with increased numbers of both alloantigen-specific Marilyn Tg and polyclonal CD4^+^ T cells in the skin (Figure 9D). T_CM_ transfer also resulted in higher frequency of transferred T cells in the spleens and mesenteric lymph nodes (Supplemental Figure 8, A–D). We also transferred polyclonal CD4^+^ T cells that contain an *Il17a*^YFP^ fate reporter to track the differentiation of Th17 cells. Interestingly, transferred T_CM_ demonstrated increased differentiation to Th17 cells in the skin as compared with T_EM_ (Figure 9E) and this was not observed in the lymphoid organs (Supplemental Figure 8, E and F). Hence, CSA-expanded CD4^+^ T_CM_ preferentially accumulate in the skin and undergo Th-17 differentiation.

## Discussion

CNIs remain the mainstay of aGVHD prophylaxis and effectively limits aGVHD, however a considerable portion of patients develop cGVHD (54). Further, CNI administration has not proven effective in the management of cGVHD other than as a potential steroid sparing agent (56). This led us to investigate the mechanisms by which CNIs modulate early alloreactive T cell immunity after BMT and subsequent effects on long-term immune tolerance. We show that although CNIs attenuate aGVHD by preventing the expansion and trafficking of effector T cells into target organs, they paradoxically suppress T cell exhaustion, expand T_CM_, and rescue alloantigen-specific CD4^+^ T cells, preventing the establishment of immune tolerance and promoting cGVHD.

Tolerance remains a uniquely double-edged sword after allogeneic BMT. An early immune response against residual malignant cells is required to provide maximal curative outcome. When the target of this donor immune response is recipient tumor or hematopoietic antigens, allogeneic BMT can provide curative graft-versus-leukemia (GVL) effects without detrimental GVHD. In practice, however, target antigens are predominantly widely expressed minor histocompatibility and/or endogenous self-antigens expressed within mismatched MHC/HLA molecules (57, 58), meaning GVL and GVHD often go hand in hand. The establishment of operational tolerance after allogeneic BMT typically is defined by the ability to remove immune suppression without the development of GVHD. This can be achieved by a number of immunological pathways that include deletion of alloreactive T cells, regulation of alloreactive T cells by regulatory T cells, and exhaustion and anergy of alloreactive T cells. IL-2 plays an important role in many of these pathways, as it is central to Treg survival and the limitation of anergy and exhaustion (59–61). In addition, nuclear factor of activated T cells (NF-AT) directly contributes to T cell exhaustion by upregulating PD-1 and TOX expression (42, 62). It is thus not surprising that CNIs, which target the NF-AT/IL-2 pathway, disrupt the generation of operational tolerance. Our results demonstrate that CNIs promote the expansion of alloreactive T_CM_ with enhanced ability to mediate late GVHD upon drug withdrawal. Mechanistically, alloreactive T cells are blocked from entering all stages of the exhaustion pathway and instead accumulate as alloantigen-specific T_CM_. Importantly, the majority of activated T cells preferentially acquire a T_EFF_ phenotype with broad upregulation of Ly6C such that this is not a reliable marker for transitory T_EX_ (or T_TRANS_-like cells) as was used in a recent study (41). Interestingly, the CNI-expanded T_CM_ population retain TCF-1 expression, consistent within their enhanced proliferative and pathogenic potential (10, 11) following CNI treatment and withdrawal. As previously demonstrated, TCF-1^hi^ T_CM_ that migrate to and reside in target tissues are also likely to maintain GVHD locally (11). Of interest, TAC was far more potent than CSA in mediating these effects, likely reflecting the described increased potency and ability of tacrolimus to inhibit additional cyclosporine-independent pathways (63). It is difficult to extrapolate the findings in relation to CNIs and alloantigen-driven CD4^+^ T cell differentiation to that invoked by viral antigen since that amount of antigen presented is quite different quantitatively and temporally (i.e., alloantigen is present in high amounts and forever but viral antigen is generally present at low levels and transiently). Nevertheless, this will be an interesting avenue of future study.

Donor T_N_ have higher ability to induce GVHD due to high TCR diversity that can respond to large numbers of alloantigen and high proliferation potential and lineage plasticity (5). T_EM_ have reduced ability to induce GVHD; they demonstrate lower capacity of survival and expansion in response to alloantigen (14, 64). In contrast, T_CM_ which are thought to be derived from surviving effector T cell pools (65), maintain the capacity to invoke and maintain GVHD (11, 14). This concept has recently been confirmed in large clinical studies of T_N_ depletion (3). Thus, the process of alloantigen driven T cell differentiation largely determines the capacity of the donor T cell pool to mediate GVHD. Here, we demonstrate that CNIs inhibit predominantly CD4^+^ T cell apoptosis and allow more donor T cells to differentiate into alloantigen-specific T_CM._ Further, CNI-induced T_CM_ demonstrate higher proliferation potential and enhanced ability to mediate late onset GVHD. Hence, alloreactive donor T cells that differentiated in the presence of a CNI were limited in their effector (e.g., cytokine) function but subsets preferentially survived to become T_CM_ that retained self-renewal ability and were capable of generating cGVHD following drug withdrawal. This detrimental effect of CNIs was most pronounced in CD4^+^ T cells since TCR sequencing also confirmed that alloreactive CD8^+^ T cell subsets were efficiently suppressed.

The similarity of phenotypic, transcriptomic, and functional changes in CD4^+^ and CD8^+^ T cells suggests common mechanisms in the T_CM_ expansion. Metabolic reprogramming is critical for the differentiation, function, and memory formation of T cells (66). Generally, high glycolytic activity is required for effector function and T cell exhaustion, which impairs long-term immunity (67). In a GVHD model, rapamycin inhibits the glycolytic activity leading to attenuation of T cell activation and subsequent GVHD development, whereas the OXPHOS pathway is not affected (37). Alternatively, OXPHOS promotes the survival and self-renewal ability of T cells in the context of antigen stimulation (68, 69), which potentially explains the functional quiescence and higher self-renewal capacity of alloreactive T cells in the presence of CNIs. Additional investigation is required to elucidate the mechanisms by which CNIs regulate T cell metabolism and differentiation and are beyond the scope of the current study. Finally, IL-2 deprivation upregulated IL-2R-β expression as a feed-back response, which theoretically increases the formation of the IL-15R and subsequent signaling. However, disruption of IL-15R signaling had no effect on CNI-induced T_CM_ expansion, and analysis of other common γ-chain cytokines such as IL-7 are needed. In contrast to the aforementioned similarities, activated CD8^+^ T cells demonstrate higher responsiveness to and dependence on exogenous IL-2 relative to CD4^+^ T cells (70). This, likely, in part, explains the relative resistance of CD4^+^ T cells to CNIs.

CNIs and PT-Cy have different mechanisms of action on alloreactive donor T cells that leads to disparate long-term outcomes. First, alloantigen-specific CD4^+^ T cells are more resistant to CNI inhibition, predisposing recipients to cGVHD following CNI discontinuation. In contrast, proliferating effector T cells are selectively deleted by PT-Cy, which allows for the eventual regeneration of donor T cells from nonalloreactive T_N_-derived T_SCM_ cells (6, 51). Second, IL-17A–producing cells (Th17 and Tc17), which are major mediators of cGVHD especially in the skin (27), have stem-like properties and can survive long-term (55, 71). While these IL-17A–producing cells are resistant to CNI treatment — and can preferentially expand in the presence of CNI in target tissues (32) — they are effectively deleted by PT-Cy. Third, PT-Cy (52, 72), unlike CNIs (25), target conventional T cells but spare Tregs, further promoting tolerance. Hence, the use of CNI after PT-Cy, when alloreactive clones are largely eliminated, is likely to avoid the detrimental effect of this agent on long-term tolerance. Indeed, PT-Cy followed by CNI was synergistic in preventing GVHD (73); however, use of CNIs prior to Cy abrogated this synergy in a mouse model of skin allotransplantation (74). It is important to note that CNIs have been historically combined with short course methotrexate (MTX) and it is likely that this antimetabolite is critical in controlling some of the detrimental effects of CNIs on alloreactive T cell reconstitution. Nevertheless, recent clinical studies demonstrate that a CNI/MTX combination is less effective than PT-Cy followed by a CNI, as would be predicted by the findings here (75).

In summary, we demonstrate that CNI-based immune suppression in the absence of approaches that eliminate large fractions of alloreactive T cell clones (such as T_N_ depletion or PT-Cy) are efficient at limiting aGVHD, but at the cost of promoting the persistence and accumulation of alloreactive T_CM_ clones that preferentially survive to mediate cGVHD. Whether the use of non-CNI based immune suppression (e.g., abatacept) after PT-Cy can further reduce the incidence of cGVHD and improve transplant outcomes warrants investigation.

## Methods

Additional methods and a list of antibodies used in the study are available in Supplemental Methods.

### Sex as a biological variant.

The preclinical murine systems utilized both female and male mice. Importantly, there is no evidence to suggest GVHD or CNI-mediated immune suppression is sex restricted.

### Mice.

C57Bl/6 (B6, H-2b, CD45.2^+^, CD90.2^+^), B6.SJL-Ptprca (PTPrca, H-2b, CD45.1^+^, CD90.2^+^), C57xPTP (H-2b, CD45.2^+^CD45.1^+^, CD90.2^+^), and B6D2F1 (H-2b/d, CD45.2^+^, CD90.2^+^) mice were purchased from the Animal Resources Centre, Jackson Laboratory, or bred in the animal facilities at Fred Hutchinson Cancer Center (FHCC). *Il15ra^–/–^* (B6x129S F2 background) and control mice were purchased from Jackson Laboratory. TEa TCR transgenic mice (*Rag1^–/–^* background, H-2b, CD45.1^+^, CD90.2^+^) (40), Marilyn TCR transgenic mice (*Rag2^–/–^* background, H-2b, CD45.2^+^, CD90.2^+^) (50), B6.TEaxB6.luc^+^ (H-2b, CD45.1^+^, CD90.1^+^), B6.MarilynxB6.luc^+^ (H-2b, CD45.2^+^, CD90.1^+^), H-Y TCR transgenic (H2b, CD45.2^+^, CD90.2^+^) (40), B6.*Il17a*^Cre^x *Rosa26*^eYFP^(B6.*Il17a*^eYFP^, H-2b, CD45.2^+^) (55), and B6.*Il10*^GFP^x*Foxp3*^RFP^ (H-2b, CD45.2^+^) (29) mice were bred and housed at QIMR Berghofer Medical Research Institute or FHCC. Mice were housed in sterilized microisolator cages and received acidified autoclaved water (pH 2.5).

### Bone marrow transplantation.

Recipients received 1,100 (B6D2F1) or 1,000 (C57BL/6) cGy total body irradiation (TBI) (^137^Cs source) on day –1 as conditioning regimen. In some experiments, recipients received 900 cGy TBI on day –1 plus 100 mg/kg cyclophosphamide on days –3 and –2. T cell-depleted (TCD) BM and CD4^+^, CD8^+^, and CD3^+^ T cells were processed as described previously (76). CSA (5–50 mg/kg/d) or TAC (FK506) (1 or 10 mg/kg/d) was administered i.p. daily from day 0 up until day 13 after BMT (14 days maximum). PT-Cy (cyclophosphamide 50 or 100 mg/kg/d) was administrated i.p. on days 3 and 4. GVHD severity was scored with a clinical scoring system as previously described (76).

### Flow cytometry.

Staining of surface markers, transcription factors, and cytokines (intracellular cytokine staining or ICS) was performed as previously described (29). Antibodies are listed in the Supplemental Methods. Single cell suspensions were acquired on LSR Fortessa or Symphony A3 flow cytometers (Becton Dickinson) and analyzed with FlowJo version 10 (Tree Star). T-SNE and FlowSOM analysis was conducted as previously described (77).

### Single cell RNA-Seq.

Donor T cells were isolated from spleens on day 7 after BMT and proceeded to single cell RNA-Seq as described previously (51, 77). More details can be found in the supplemental methods. Code is accessible at https://github.com/furlan-lab/CSA_GVHD (Commit ID:e87f86c).

### Statistics.

Statistical analyses were performed using GraphPad Prism version 8 (GraphPad Software). The Mann-Whitney *U* test or 2-tailed *t* test was used to determine the differences between groups. Multiple comparisons were conducted with 1-way ANOVA or 2-way ANOVA where appropriate. The log-rank test was used for the comparison of survival curves. A 2-sided *P* value < 0.05 was considered statistically significant.

### Study approval.

All animal experiments were approved by and performed in accordance with the Animal Ethics Committee at the QIMR Berghofer Medical Research Institute or the IACUC of the Fred Hutchinson Cancer Center, where experimentation was undertaken.

### Data availability.

Original data are available in the supporting data file. RNA-Seq data have been deposited in National Center for Biotechnology Information (NCBI) Gene Expression Omnibus (GEO) with accession number GSE255545.

## Author contributions

YW and MAU performed experiments, analyzed data and edited the manuscript. OGW, SSB and SAM analyzed RNA sequencing data and edited the manuscript. NSN, KSE, CRS, SRWL, TS, RDK, ELN, EA, ACY, ST, JRB, AV, BRB and MK helped with experimental design and edited the manuscript. ADC conducted the histopathologic scoring and edited the manuscript. SNF oversaw the RNA sequencing analysis and edited the manuscript. PZ designed and performed experiments, conducted and oversaw data analysis, and wrote the manuscript. GRH supervised the research and wrote the manuscript.

## Supplementary Material

Supplemental data

Supporting data values

## Version 1 06/03/2024

Electronic publication

## References

[1] Zeiser R, Blazar BR (2017). Acute graft-versus-host disease - biologic process, prevention, and therapy. N Engl J Med.

[2] Zeiser R, Blazar BR (2017). Pathophysiology of chronic graft-versus-host disease and therapeutic targets. N Engl J Med.

[3] Bleakley M (2022). Naive T cell depletion to prevent chronic graft-versus-host disease. J Clin Oncol.

[4] Anderson BE (2003). Memory CD4^+^ T cells do not induce graft-versus-host disease. J Clin Invest.

[5] Hataye J (2006). Naive and memory CD4^+^ T cell survival controlled by clonal abundance. Science.

[6] Roberto A (2015). Role of naive-derived T memory stem cells in T cell reconstitution following allogeneic transplantation. Blood.

[7] Cieri N (2015). Generation of human memory stem T cells after haploidentical T-replete hematopoietic stem cell transplantation. Blood.

[8] Zhou X (2010). Differentiation and persistence of memory CD8^+^ T cells depend on T cell factor 1. Immunity.

[9] Siddiqui I (2019). Intratumoral Tcf1^+^PD-1^+^CD8^+^ T cells with stem-like properties promote tumor control in response to vaccination and checkpoint blockade immunotherapy. Immunity.

[10] Tsui C (2022). MYB orchestrates T cell exhaustion and response to checkpoint inhibition. Nature.

[11] Sacirbegovic F (2023). Graft-versus-host disease is locally maintained in target tissues by resident progenitor-like T cells. Immunity.

[12] Zhang Y (2005). Alloreactive memory T cells are responsible for the persistence of graft-versus-host disease. J Immunol.

[13] Zheng H (2009). Central memory CD8^+^ T cells induce graft-versus-host disease and mediate graft-versus-leukemia. J Immunol.

[14] Juchem KW (2011). A repertoire-independent and cell-intrinsic defect in murine GVHD induction by effector memory T cells. Blood.

[15] Graef P (2014). Serial transfer of single-cell-derived immunocompetence reveals stemness of CD8^+^ central memory T cells. Immunity.

[16] Stemberger C (2014). Lowest numbers of primary CD8^+^ T cells can reconstitute protective immunity upon adoptive immunotherapy. Blood.

[17] Zhang Y (2004). Dendritic cell-activated CD44^hi^CD8^+^ T cells are defective in mediating acute graft-versus-host disease but retain graft-versus-leukemia activity. Blood.

[18] Malek TR (2008). The biology of interleukin-2. Annu Rev Immunol.

[19] Kalia V (2010). Prolonged interleukin-2Ralpha expression on virus-specific CD8+ T cells favors terminal-effector differentiation in vivo. Immunity.

[20] Castro I (2011). The basis of distinctive IL-2- and IL-15-dependent signaling: weak CD122-dependent signaling favors CD8+ T central-memory cell survival but not T effector-memory cell development. J Immunol.

[21] Chow CW (1999). Requirement for transcription factor NFAT in interleukin-2 expression. Mol Cell Biol.

[22] Emmel EA (1989). Cyclosporin A specifically inhibits function of nuclear proteins involved in T cell activation. Science.

[23] Hamilton BK (2018). Current approaches to prevent and treat GVHD after allogeneic stem cell transplantation. Hematology Am Soc Hematol Educ Program.

[24] Jenkins MK (1988). Effects of cyclosporine A on T cell development and clonal deletion. Science.

[25] Zeiser R (2006). Inhibition of CD4^+^CD25^+^ regulatory T cell function by calcineurin-dependent interleukin-2 production. Blood.

[26] Luznik L (2012). Post-transplantation cyclophosphamide for tolerance induction in HLA-haploidentical bone marrow transplantation. Semin Oncol.

[27] Hill GR (2010). Stem cell mobilization with G-CSF induces type 17 differentiation and promotes scleroderma. Blood.

[28] Alexander KA (2014). CSF-1-dependant donor-derived macrophages mediate chronic graft-versus-host disease. J Clin Invest.

[29] Zhang P (2017). Eomesodermin promotes the development of type 1 regulatory T (T_R_1) cells. Sci Immunol.

[30] Reagan-Shaw S (2008). Dose translation from animal to human studies revisited. FASEB J.

[31] Blanchard OL, Smoliga JM (2015). Translating dosages from animal models to human clinical trials--revisiting body surface area scaling. FASEB J.

[32] Varelias A (2015). Lung parenchyma-derived IL-6 promotes IL-17A-dependent acute lung injury after allogeneic stem cell transplantation. Blood.

[33] el Mansour A (1996). Cyclosporin depresses pancreatic islet expression of antigens for islet cell autoantibodies in non obese diabetic mice. J Autoimmun.

[34] Ewart SL (1996). Cyclosporin A attenuates genetic airway hyperresponsiveness in mice but not through inhibition of CD4+ or CD8+ T cells. Am J Respir Cell Mol Biol.

[35] Okamoto T (1999). The protective effect of cyclosporine A on anti-Fas antibody-induced hepatitis in mice. Jpn J Pharmacol.

[36] Gartlan KH (2017). Th17 plasticity and transition toward a pathogenic cytokine signature are regulated by cyclosporine after allogeneic SCT. Blood Adv.

[37] Nguyen HD (2016). Metabolic reprogramming of alloantigen-activated T cells after hematopoietic cell transplantation. J Clin Invest.

[38] Shi LZ (2011). HIF1alpha-dependent glycolytic pathway orchestrates a metabolic checkpoint for the differentiation of TH17 and Treg cells. J Exp Med.

[39] van der Windt GJ, Pearce EL (2012). Metabolic switching and fuel choice during T cell differentiation and memory development. Immunol Rev.

[40] Koyama M (2015). Donor colonic CD103^+^ dendritic cells determine the severity of acute graft-versus-host disease. J Exp Med.

[41] Senjo H (2023). Calcineurin inhibitor inhibits tolerance induction by suppressing terminal exhaustion of donor T cells after allo-HCT. Blood.

[42] Scott AC (2019). TOX is a critical regulator of tumour-specific T cell differentiation. Nature.

[43] Khan O (2019). TOX transcriptionally and epigenetically programs CD8^+^ T cell exhaustion. Nature.

[44] Seo H (2019). TOX and TOX2 transcription factors cooperate with NR4A transcription factors to impose CD8^+^ T cell exhaustion. Proc Natl Acad Sci U S A.

[45] Alfei F (2019). TOX reinforces the phenotype and longevity of exhausted T cells in chronic viral infection. Nature.

[46] Yao C (2019). Single-cell RNA-seq reveals TOX as a key regulator of CD8^+^ T cell persistence in chronic infection. Nat Immunol.

[47] Hudson WH (2019). Proliferating transitory T Cells with an effector-like transcriptional signature emerge from PD-1^+^ stem-like CD8^+^ T cells during chronic infection. Immunity.

[48] Li H (2019). Dysfunctional CD8 T cells form a proliferative, dynamically regulated compartment within human melanoma. Cell.

[49] Kurtulus S (2019). Checkpoint blockade immunotherapy induces dynamic changes in PD-1^–^CD8^+^ tumor-infiltrating T cells. Immunity.

[50] Koyama M (2019). MHC class II antigen presentation by the intestinal epithelium initiates graft-versus-host disease and is influenced by the microbiota. Immunity.

[51] Minnie SA (2022). Depletion of exhausted alloreactive T cells enables targeting of stem-like memory T cells to generate tumor-specific immunity. Sci Immunol.

[52] Ganguly S (2014). Donor CD4^+^ Foxp3^+^ regulatory T cells are necessary for posttransplantation cyclophosphamide-mediated protection against GVHD in mice. Blood.

[53] MacDonald KP (2017). Cytokine mediators of chronic graft-versus-host disease. J Clin Invest.

[54] MacDonald KP (2017). Chronic graft-versus-host disease: biological insights from preclinical and clinical studies. Blood.

[55] Gartlan KH (2015). Tc17 cells are a proinflammatory, plastic lineage of pathogenic CD8^+^ T cells that induce GVHD without antileukemic effects. Blood.

[56] Koc S (2002). Therapy for chronic graft-versus-host disease: a randomized trial comparing cyclosporine plus prednisone versus prednisone alone. Blood.

[57] Koyama M, Hill GR (2019). The primacy of gastrointestinal tract antigen-presenting cells in lethal graft-versus-host disease. Blood.

[58] Koyama M, Hill GR (2016). Alloantigen presentation and graft-versus-host disease: fuel for the fire. Blood.

[59] Hartigan CR (2019). Memory T cell exhaustion and tolerance in transplantation. Immunol Rev.

[60] Blazar BR (2003). Blockade of programmed death-1 engagement accelerates graft-versus-host disease lethality by an IFN-gamma-dependent mechanism. J Immunol.

[61] Liu Y (2021). IL-2 regulates tumor-reactive CD8^+^ T cell exhaustion by activating the aryl hydrocarbon receptor. Nat Immunol.

[62] Oestreich KJ (2008). NFATc1 regulates PD-1 expression upon T cell activation. J Immunol.

[63] Almawi WY, Melemedjian OK (2000). Clinical and mechanistic differences between FK506 (tacrolimus) and cyclosporin A. Nephrol Dial Transplant.

[64] Anderson BE (2008). Effects of donor T cell trafficking and priming site on graft-versus-host disease induction by naive and memory phenotype CD4 T cells. Blood.

[65] Lanzavecchia A, Sallusto F (2005). Understanding the generation and function of memory T cell subsets. Curr Opin Immunol.

[66] Almeida L (2016). Metabolic pathways in T cell activation and lineage differentiation. Semin Immunol.

[67] Sukumar M (2013). Inhibiting glycolytic metabolism enhances CD8^+^ T cell memory and antitumor function. J Clin Invest.

[68] Vardhana SA (2020). Impaired mitochondrial oxidative phosphorylation limits the self-renewal of T cells exposed to persistent antigen. Nat Immunol.

[69] Hong HS (2022). OXPHOS promotes apoptotic resistance and cellular persistence in T_H_17 cells in the periphery and tumor microenvironment. Sci Immunol.

[70] Au-Yeung BB (2017). IL-2 Modulates the TCR signaling threshold for CD8 but not CD4 T cell proliferation on a single-cell level. J Immunol.

[71] Muranski P (2011). Th17 cells are long lived and retain a stem cell-like molecular signature. Immunity.

[72] Ikegawa S (2019). PTCy ameliorates GVHD by restoring regulatory and effector T cell homeostasis in recipients with PD-1 blockade. Blood Adv.

[73] Ruggeri A (2018). Post-transplant cyclophosphamide for graft-versus-host disease prophylaxis in HLA matched sibling or matched unrelated donor transplant for patients with acute leukemia, on behalf of ALWP-EBMT. J Hematol Oncol.

[74] Nomoto K (1992). Interference with cyclophosphamide-induced skin allograft tolerance by cyclosporin A. J Immunol.

[75] Holtan SG (2022). Post-transplant cyclophosphamide, tacrolimus, and mycophenolate mofetil as the new standard for graft-versus-host disease (GVHD) prophylaxis in reduced intensity conditioning: results from phase III BMT CTN 1703. Blood.

[76] Zhang P (2013). Induced regulatory T cells promote tolerance when stabilized by rapamycin and IL-2 in vivo. J Immunol.

[77] Minnie SA (2023). TIGIT inhibition and lenalidomide synergistically promote antimyeloma immune responses after stem cell transplantation in mice. J Clin Invest.

