# Supplementary material for: Calcineurin inhibition rescues alloantigen-specific central memory T cell subsets that promote chronic GVHD
Source: J Clin Invest. 2024 Jun 3;134(11):e170125. doi: 10.1172/JCI170125 (PMC11142741; doi:10.1172/JCI170125)
Supplement: Supplemental data [file jci-134-170125-s161.pdf]

Supplementary methods

Single cell RNA sequencing and data analysis. Spleens were taken on day 7 after BMT, pooled from every 2 mice and labelled with oligo-conjugated hashtag antibodies (HTO) (TotalSeq C, BioLegend) in addition to antibodies against plus CD4, CD8 and CD90.2. Cells were subsequently sort purified to $CD90.2^+CD4^+CD8^-$ or $CD90.2^+CD4^-CD8^+$ cells and captured on 10X Genomics Chromium platform with 5-prime VDJ-enrichment chemistry. Samples from same group were combined (with equal number of cells per sample) before RNA library preparation. All libraries were sequenced on NextSeq 2000 (Illumina P3 kit) targeting 20,000 reads per cell for gene expression and 5,000 reads per cell for TCR libraries. Illumina BCL reads were demultiplexed and prepared using cellranger multi. HTO reads were used to identify mice and experimental conditions after removing cells with reads for more than one HTO. The remaining cells were filtered using the following parameters: 3 < log RNA < 5; percent mitochondrial RNA < 15%. CD4 and CD8 cells were subsetted using UMI counts for both protein and RNA levels of CD4 and CD8. Batch effects were corrected using RPCA.[1] TEa cells were identified by their expression of CD45.1 and their monoclonal expression of TCR containing the CDR3 amino acid sequence CAASRNSGTYQRF_CASSTGSSYEQYF and excluded from downstream TCR analysis. Marker genes using a standard Seurat workflow were identified for each cluster and cell types were assigned using expert annotation. Metabolic gene sets were sourced from MSigDB .T_{EX} and T_{SCM} gene sets were sourced from previously published work.[2] Gene set scores were made using the 'AddModuleScore' function in Seurat. TCR analysis was performed using the package scRepertoire.[3]

Quantification of trough levels of CSA and TAC. Whole blood was collected into K3EDTA vials on day +5 after BMT before the next dose and tested at Department of Laboratory Medicine and Pathology of UW Medicine (University of Washington, Seattle, WA). The concentration of CSA and TAC in whole blood was determined by liquid chromatography-tandem mass spectrometry after protein precipitation. The reportable range was 15 – 6500 ng/mL for CSA and 1.0 – 195 ng/mL for TAC.

Isolation of skin infiltrating lymphocytes. Skin infiltrating lymphocytes were isolated from skin tissues with digestion followed by mechanic disruption. Briefly, sliced skin tissues were incubated in Hank's buffer for 2 hours at 37 °C in the presence of Collagenase type 3 (1 mg/mL; Worthington),

DNase I (100 µg/mL; Worthington) and 2% FCS before pushing through 70 – 100 µM filters. Filter-though was harvested, washed and used for flow cytometric analysis.

In vivo and organ luminescence imaging. Expansion of TEa^{luc+} and Marilyn^{luc+} T cells was determined with Xenogen imaging system (Xenogen IVIS 100; PerkinElmer) as previously described.[4] Data were analyzed with Living Image software version 4 (PerkinElmer).

Histology. Hematoxylin and eosin (HE) stained sections of skin were processed as previously described and examined in a blinded fashion (by A.D.C).[4]

Antibodies

Antibody	Clone	Fluorochromes	Catalog #	Suppliers
CD3	145-2C11	PE/Cy7	100320	Biolegend
CD3	145-2C11	PE/Cy5	100310	Biolegend
CD3	145-2C11	BV711	100349	Biolegend
CD90.2 (Thy1.2)	53-2.1	BV605	140318	Biolegend
CD90.2 (Thy1.2)	53-2.1	BV480	566075	BD Bioscience
CD90.1 (Thy1.1)	HIS51	APC-eFluor 780	47-0900-82	eBioscience
CD90.1 (Thy1.1)	HIS51	BUV805	741974	BD Bioscience
CD4	GK1.5	AF700	100430	Biolegend
CD4	GK1.5	BUV496	612952	BD Bioscience
CD4	RM4-5	Pacific Blue	100531	Biolegend
CD4	GK1.5	BV786	100453	Biolegend
CD8	53-6.7	PerCP/Cy5.5	100710	Biolegend
CD8	53-6.7	APC/Cy7	100714	Biolegend
CD8	53-6.7	BUV805	612898	BD Bioscience
CD45	30-F11	BUV395	564279	BD Bioscience
CD45.1	A20	APC/Cy7	110716	Biolegend
CD45.1	A20	BUV395	565212	BD Bioscience
CD45.2	104	FITC	109806	Biolegend
CD45.2	104	AF700	109822	Biolegend
H2Dd	34-2-12	FITC	110606	Biolegend
H2Dd-biotin	34-2-12	N/A	110606	Biolegend
H2Db	KH95	PE	111508	Biolegend
CD44	IM7	APC/Cy7	103028	Biolegend
CD62L	MEL-14	AF700	104426	Biolegend
CD62L	MEL-14	PerCP/Cy5.5	104432	Biolegend
CD62L	MEL-14	BV480	746726	BD Bioscience
CD62L	MEL-14	PE	104408	Biolegend
CD62L	Mel-14	BUV737	612833	BD Bioscience
TCR Vα2	B20.1	APC/Cy7	127818	Biolegend
TCR Vβ6	RR4-7	FITC	553193	BD Bioscience
Ly6C	AL-21	FITC	561085	BD Bioscience

Ly6C	AL-21	PE/Dazzle 594	128044	Biolegend
CD122	TMb1	PerCP-eFluor 710	46-1222-82	eBioscience
KLRG1	2F1	BV786	565477	BD Bioscience
PD-1 (CD279)	J43	BUV737	568362	BD Bioscience
Ly108	13G3	BUV661	741679	BD Bioscience
CD69	H1.2F3	BUV563	741234	BD Bioscience
Tim-3	RMT3-23	BV605	119721	Biolegend
CX3CR1	SA011F11	FITC	149020	Biolegend
CX3CR1	SA011F11	AF647	149004	Biolegend
CD226 (DNAM-1)	TX42.1	BV650	TX42.1	Biolegend
TIGIT	IG9	BV421	142111	Biolegend
T-bet	4-B10	AF647	644804	Biolegend
BCL-2	BCL/10C4	AF647	633510	Biolegend
Caspase-3	C92-605	PE	550821	BD Bioscience
FoxP3	150D	AF647	320014	Biolegend
FoxP3	FJK-16s	PE/Cy5	15-5773-82	eBioscience
Ki-67	16A8	PE/Cy7	652426	Biolegend
TOX	TXRX10	eFluor 660	50-6502-82	eBioscience
Eomes	Dan11mag	PE/Cy7	25-4875-82	eBioscience
TCF-7/TCF-1	S33-966	PE	564217	BD Bioscience
Granzyme B	QA16A02	PE/Dazzle 594	372216	Biolegend
Granzyme B	GB11	AF700	560213	BD Bioscience
IL-10	JES6-16E3	PE	505008	Biolegend
IFNγ	XMG1.2	BV421	505830	Biolegend
IFNγ	XMG1.2	BV785	505838	Biolegend
TNF	MP6-XT22	BB700	566510	BD Bioscience
TNF	MP6-XT22	PE	506306	Biolegend
TNF	MP6-XT22	APC	506308	Biolegend
IL-17A	TC11-8H10.1	AF700	506914	Biolegend
CD16/CD32 (Fc	2.4G2	N/A	553142	BD Bioscience

References

1. Hao Y, Hao S, Andersen-Nissen E, et al. Integrated analysis of multimodal single-cell data. *Cell*. 2021;184(13):3573-3587.e3529.
2. Minnie SA, Waltner OG, Ensbey KS, et al. Depletion of exhausted alloreactive T cells enables targeting of stem-like memory T cells to generate tumor-specific immunity. *Sci Immunol*. 2022;7(76):eabo3420.
3. Borcherding N, Bormann NL, Kraus G. scRepertoire: An R-based toolkit for single-cell immune receptor analysis. *F1000Res*. 2020;9:47.
4. Zhang P, Tey SK, Koyama M, et al. Induced regulatory T cells promote tolerance when stabilized by rapamycin and IL-2 in vivo. *J Immunol*. 2013;191(10):5291-5303.

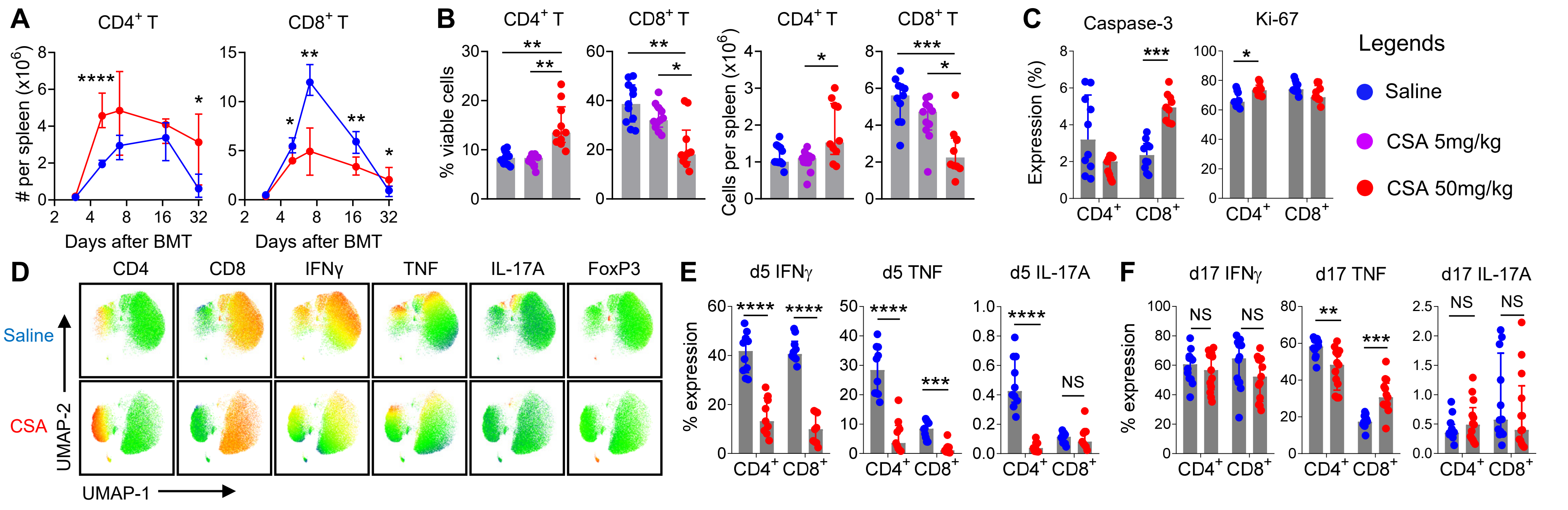

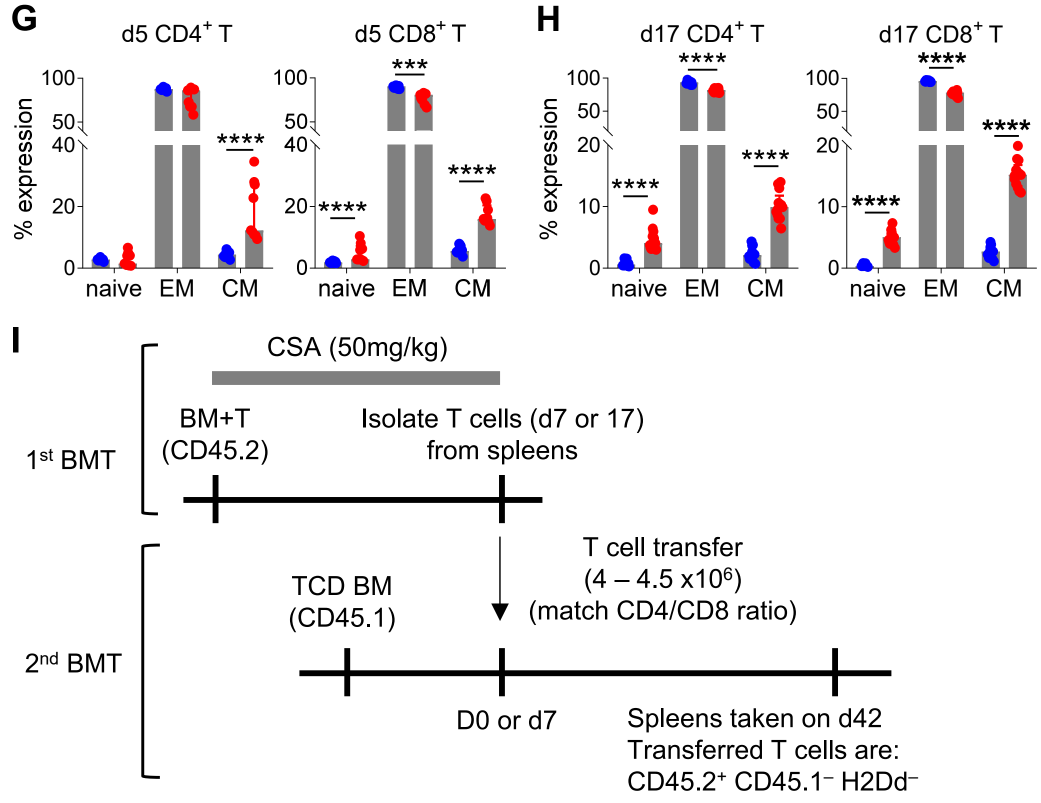

Figure S1. Effect of high dose CSA on the expansion, function and persistence of donor T cells. B6D2F1 recipients were transplanted with B6 BM ($5x10^6$) + T cells ($2x10^6$) and treated with saline or CSA (5 or 50 mg/kg). (A) Numbers of donor T cells per spleen over time after BMT. (B) Frequency and numbers of donor T cells in the spleens on day 7 (*n* = 10 – 11 per group from 2 experiments). (C) Expression of caspase-3 and Ki-67 in splenic donor T cells on day 5 prior to normalization. (D) UMAP plots (concatenated from 5 samples per group) showing the expression of cytokines and FoxP3 in donor T cells (day 5). (E, F) Expression of cytokines in donor T cells on day 5 (E, n = 8 – 9 per group from 2 experiments) and day 17 (F, *n* = 12 – 13 per group from 3 experiments). (G, H) Frequency of T_N ($CD44^-CD62L^+$), T_{EM} ($CD44^+CD62L^-$) and T_{CM} ($CD44^+CD62L^+$) subsets in donor T cells on day 5 (G) and day 17 with representative flow cytometric plots (H, n = 9 – 13 per group from 2 experiments). (I) Schema of adoptive transfer and analysis for saline or CSA pre-treated donor T cells. Data are presented as median ± interquartile range and analyzed with the Mann-Whitney U test (A, C – H) or one-way ANOVA (B). NS, not significant; *P < 0.05; **P < 0.01; ***P < 0.001; ****P < 0.0001.

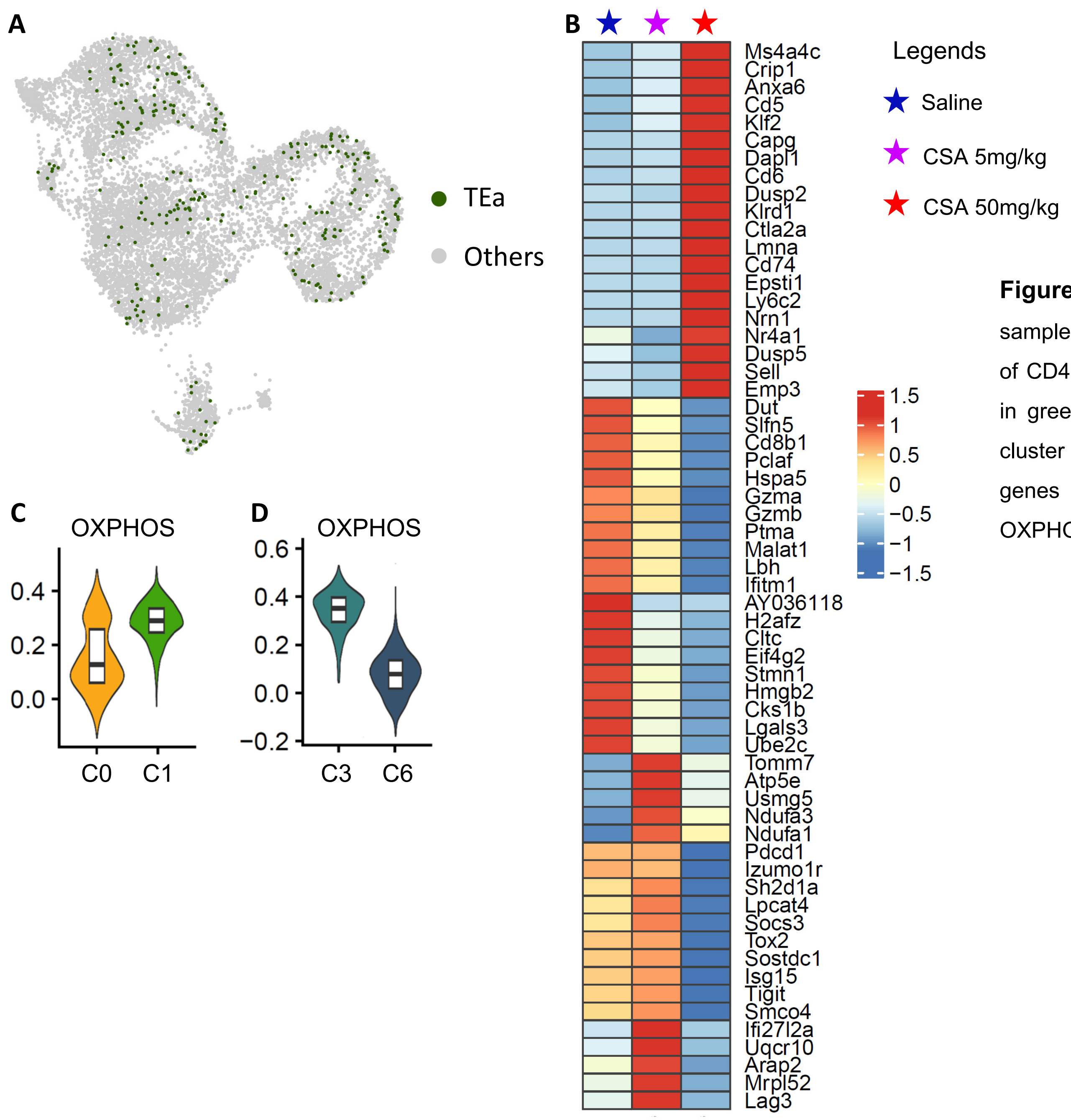

Figure S2. Single cells RNAseq in CD4⁺ T cells. The samples were processed as described in Figure 3. (A) UMAP plot of CD4⁺ T cells showing the distribution of TEa cells (highlighted in green). (B) Heatmap of top differentially expressed genes in cluster 2 across groups. (C) Expression of OXPHOS-related genes between cluster 0 and cluster 1. (D) Expression of OXPHOS-related genes between cluster 3 and cluster 6.

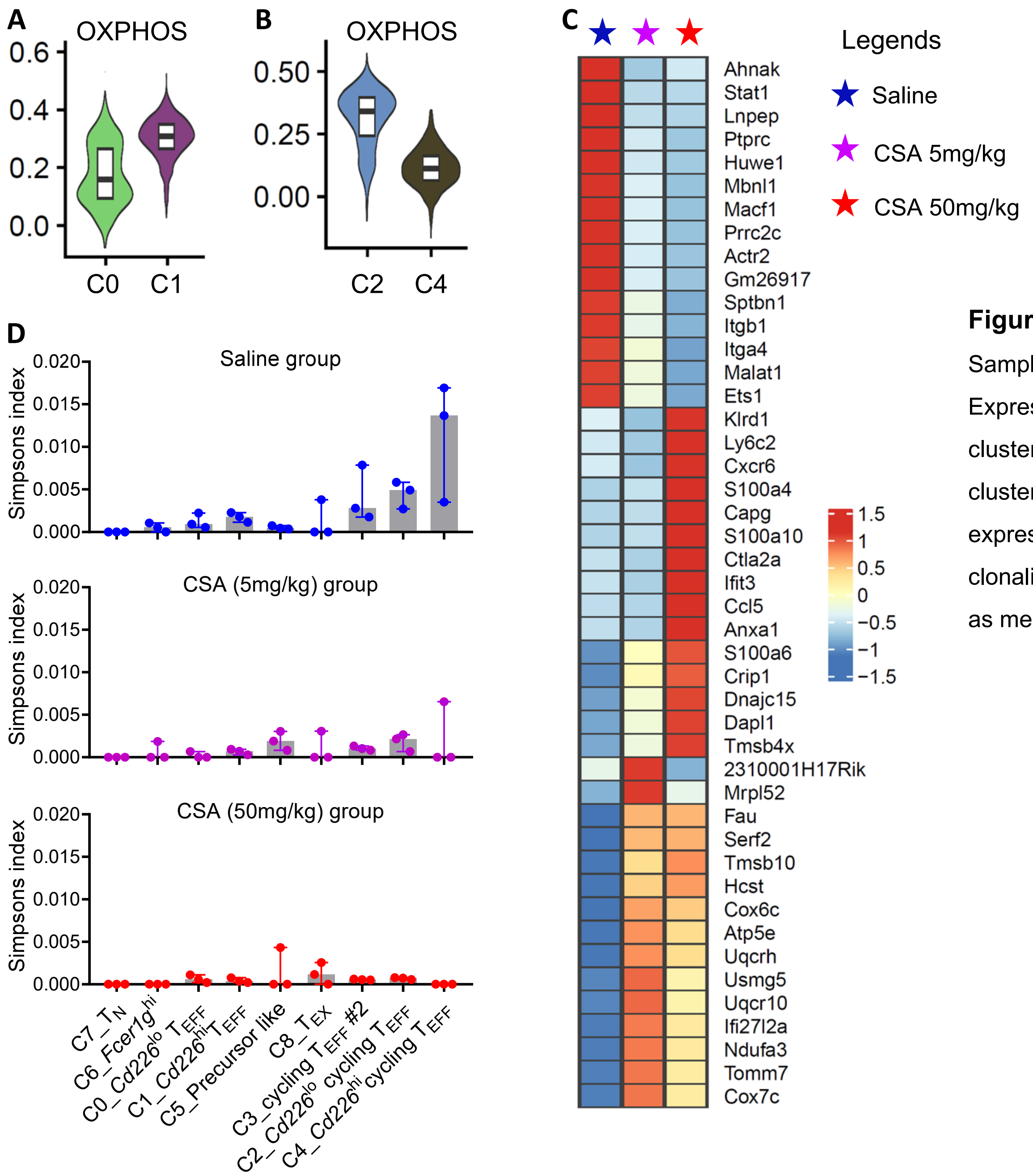

Figure S3. Single cells RNAseq in CD8⁺ T cells. Samples were processed as described in Figure 4. (A) Expression of OXPHOS-related genes between cluster 0 and cluster 1. (B) Expression of OXPHOS-related genes between cluster 2 and cluster 4. (C) Heatmap of top differentially expressed genes in cluster 5 across groups. (D) Simpson's clonality index of individual clusters across groups (presented as median ± interquartile range).

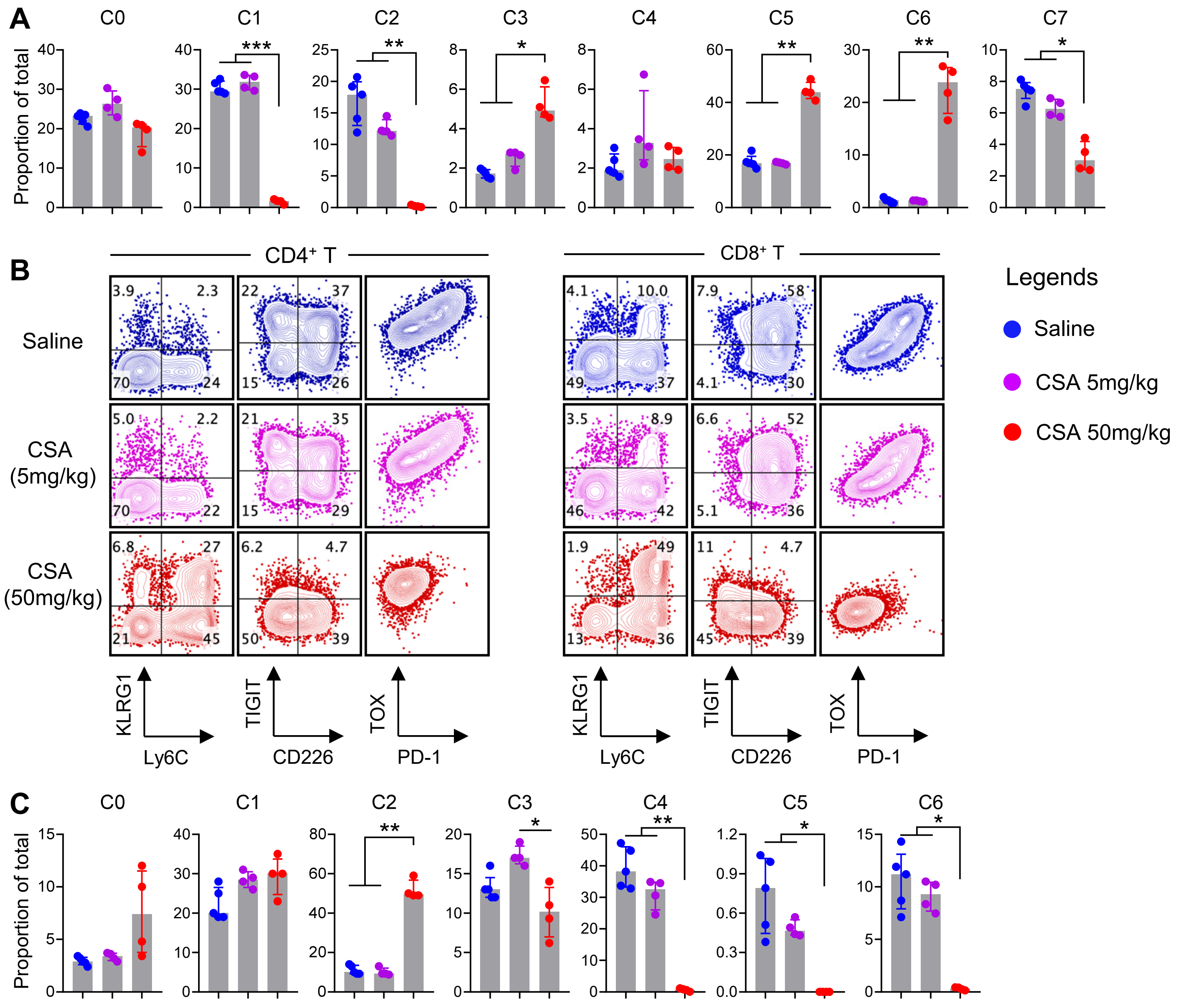

Figure S4. High dose CSA changed immune phenotypes of donor T cells. Samples were processed as described in Figure 5A – F. (A) Proportion of the FlowSOM clusters in $CD4^+$ T cells across individual samples (associated with Figure 5A). (B) Flow cytometric plots showing the expression of activation, memory and co-inhibitory markers. (C) Proportion of the FlowSOM clusters in $CD8^+$ T cells across individual samples (associated with Figure 5D). Data are presented as median ± interquartile range and analyzed with one-way ANOVA. *P < 0.05; **P < 0.01; ***P < 0.001.

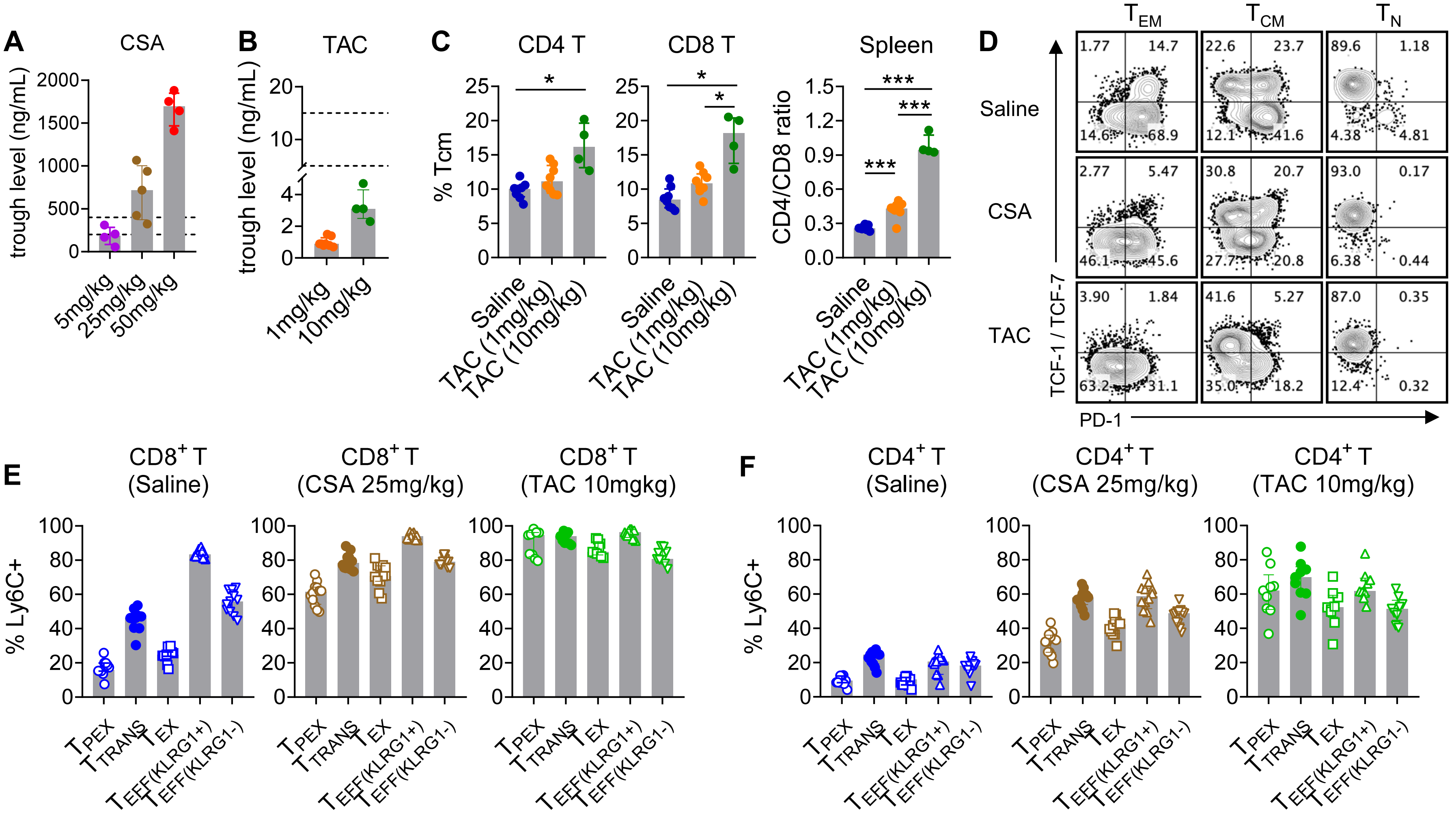

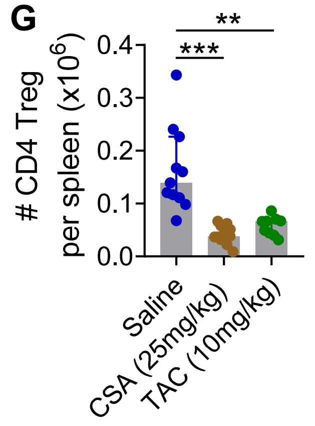

Figure S5. CSA and TAC mediate similar immunomodulatory effects on T cell differentiation. Female B6D2F1 recipients were transplanted with BM ($5x10^6$) and T cells ($2x10^6$) from B6 donors and treated with saline, CSA (5, 25 or 50mg/kg/d) or TAC (1 or 10mg/kg/d). (A – B) Whole blood was collected on day +5 and determined for the trough levels of CSA (A, n = 4 – 5 per group from 1 experiment) and TAC (B, n = 4 – 8 per group from 2 experiments). The dashed lines indicate general therapeutic ranges. (C) Spleens were taken on day +7 and analyzed for the frequency of T_{CM} in donor T cells with ratio of $CD4^+$ to $CD8^+$ T cells (n = 8, 8, 4 per group from 2 experiments). (D – G) Recipients were treated with saline, CSA (25mg/kg/d) or TAC (10mg/kg/d) from day 0. Spleens were taken for analysis on day +14 and analyzed for (D) Expression of TCF-1/TCF-7 and PD-1 in T_{EM}, T_{CM} and T_N subsets of $CD8^+$ T cells. The flow cytometric plots are concatenated from 4 – 5 samples per groups. (E) expression of Ly6C in subsets of $CD8^+$ T cells, (F) expression of Ly6C in subsets of $CD4^+$ T cells and (G) absolute numbers of $CD4^+$ $FoxP3^+$ Tregs. E – G: n = 9 – 12 per group from 2 experiments. Data are presented as median ± interquartile range and analyzed with one-way ANOVA. *$P < 0.05$; **$P < 0.01$; ***$P < 0.001$.

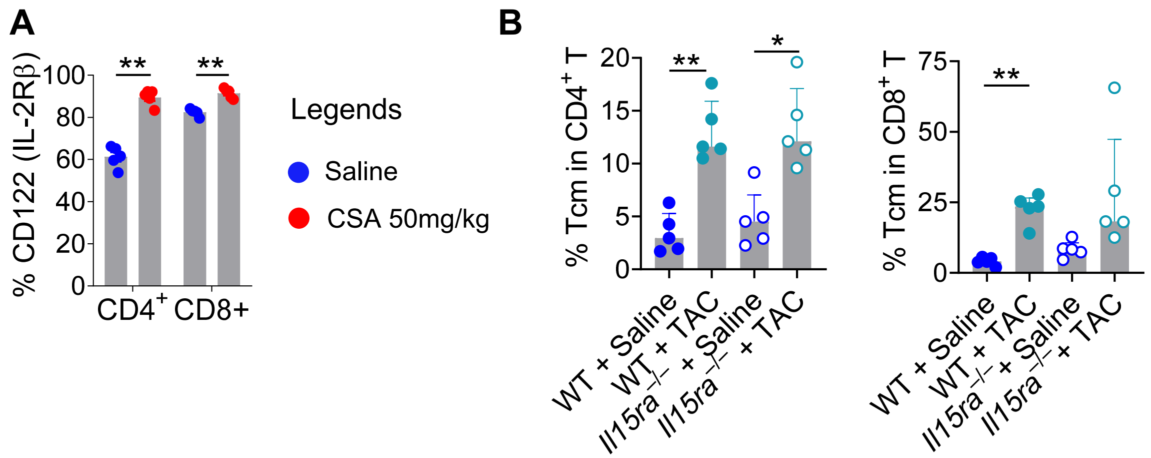

Figure S6. CNI-induced T_{CM} expansion is not dependent on IL-15R signaling. (A) Female B6D2F1 recipients were transplanted with BM ($5x10^6$) and T cells ($2x10^6$) from B6 donors and treated with saline or CSA (50mg/kg/d). Spleens were taken on day +5 and analyzed for the expression of CD122 (IL-2Rβ) on donor $CD4^+$ and $CD8^+$ T cells (n = 5 per group from 1 experiment). (B) Female B6D2F1 recipients were transplanted with BM ($5x10^6$) and T cells ($1x10^6$) from *Il15ra*$^{-/-}$ or wild-type B6/129S-F2 donors and treated with saline or TAC (10mg/kg/d) from day 0. Spleens were taken on day +14 and analyzed for the frequency of T_{CM} in donor $CD4^+$ and $CD8^+$ T cells (*n* = 5 per group from 1 experiment). Data are presented as median ± interquartile range and analyzed with the Mann-Whitney U test (A) or one-way ANOVA (B). NS, not significant; *P < 0.05; **P < 0.01.

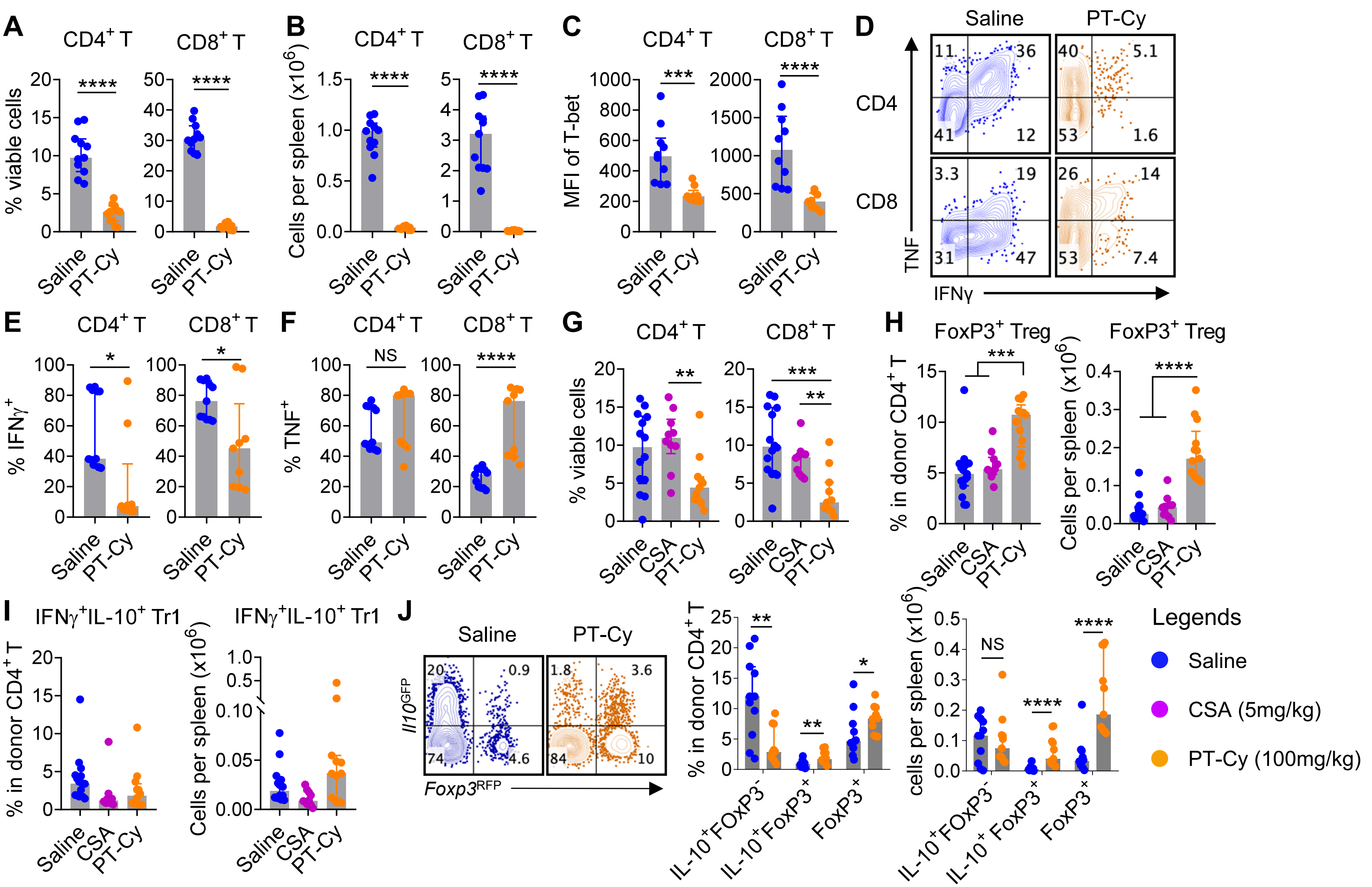

Figure S7. PT-Cy deleted activated T cells and was associated with Treg expansion late after BMT. (A – I) B6D2F1 recipients were transplanted with BM ($5x10^6$) and T cells ($2x10^6$) from B6.*Il17a*^{eYFP} donors and treated with saline, CSA (5mg/kg) or PT-Cy. (A – F) Spleens were taken on day 7 and donor T cells were analyzed (n = 9 – 11 per group from 2 experiments) for: (A) frequency in all viable cells, (B) absolute numbers per spleen, (C) expression of T-bet, (D) flow cytometric plots for IFNγ/TNF (concatenated from 5 samples per group), (E) expression of IFNγ and (F) expression of TNF. (G – I) Spleens were taken on day 30 and donor T cells were analyzed (n = 10 – 15 per group from 2 – 3 experiments) for: (G) frequency of donor T cells in all viable cells, (H) frequency and numbers of FoxP3$^+$ Tregs and (I) frequency and numbers of IFNγ$^+$IL-10$^+$ Tr1 cells (following intracellular staining). (J) B6D2F1 recipients were transplanted with BM ($5x10^6$) from Ptprca and T cells ($2x10^6$) from B6.*Foxp3*^{RFP} x *Il10*^{GFP} donors and treated with saline or PT-Cy (n = 11 per group from 2 experiments). Spleens were taken on day 30 and analyzed for the expression of IL-10 and FoxP3 (with GFP and RFP respectively) in the transferred T cells. Data are presented as median ± interquartile range and analyzed with the Mann-Whitney U test (A – F, J) or one-way ANOVA (G – I). NS, not significant; *$P < 0.05$; **$P < 0.01$; ***$P < 0.001$; ****$P < 0.0001$.

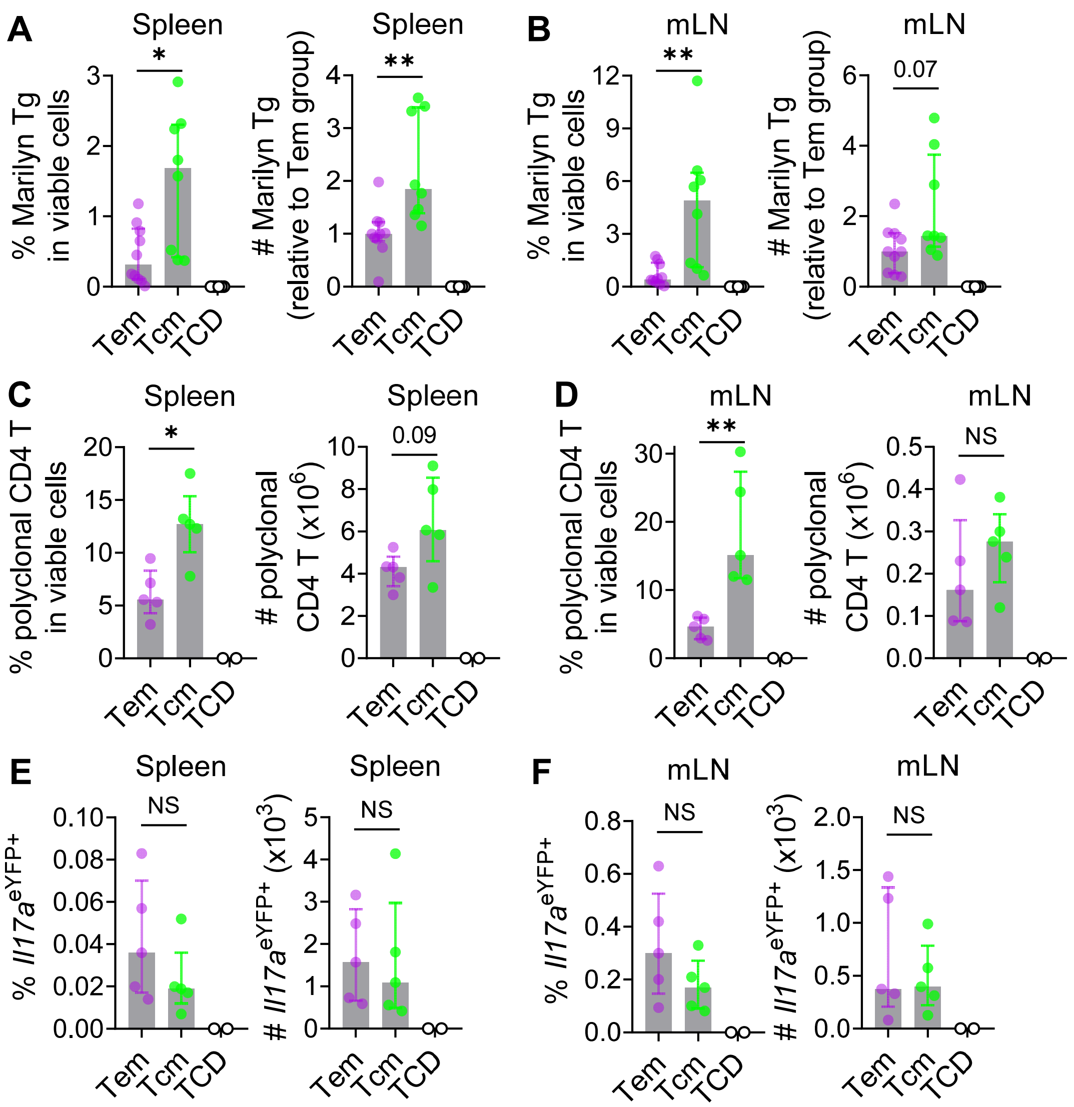

Figure S8. Alloreactive CD4⁺ T_{CM} preferentially expand after secondary transplantation. Experiments were conducted as described in Figure 9. Spleen and mesenteric lymph nodes (mLN) were analyzed with high-parameter flow cytometry whereby transferred T cells were identified with congenic markers. ((A – B) Frequency and numbers (data are normalized to the T_{EM} group) of transferred Marilyn Tg cells in the spleens and mLN. (C – D) Frequency and numbers of the transferred polyclonal CD4⁺ T cells in the spleens and mLN. (E – F) Frequency of *Il17a*$^{eYFP+}$ cells in the transferred polyclonal CD4⁺ T cells with absolute numbers in the spleens and mLN. A – B: n = 10, 8, 6 per group from 2 experiments; C – F: n = 5, 5, 2 per group from 1 experiment. Data are presented as median ± interquartile range and analyzed with the Mann-Whitney U test. NS, not significant; *P < 0.05; **P < 0.01.
